# NOD-like receptor signaling and inflammasome-related pathways are highlighted in psoriatic epidermis

**DOI:** 10.1038/srep22745

**Published:** 2016-03-15

**Authors:** Mari H. Tervaniemi, Shintaro Katayama, Tiina Skoog, H. Annika Siitonen, Jyrki Vuola, Kristo Nuutila, Raija Sormunen, Anna Johnsson, Sten Linnarsson, Sari Suomela, Esko Kankuri, Juha Kere, Outi Elomaa

**Affiliations:** 1 Folkhälsan Institute of Genetics, Helsinki, Finland; 2 Department of Medical and Clinical Genetics, Medicum and Research Programs Unit, Molecular Neurology, University of Helsinki, Helsinki, Finland; 3 Department of Biosciences and Nutrition, Karolinska Institutet, Huddinge, Sweden; 4 Science for Life Laboratory, Solna, Sweden; 5 Helsinki Burn Center, Department of Plastic Surgery, University of Helsinki and Helsinki University Hospital, Helsinki, Finland; 6 Department of Pharmacology, Medicum, University of Helsinki, Helsinki, Finland; 7 Biocenter Oulu, Department of Pathology, University of Oulu, Oulu, Finland; 8 Department of Medical Biochemistry and Biophysics, Karolinska Institutet, Stockholm, Sweden; 9 Department of Dermatology, University of Helsinki and Helsinki University Hospital, Helsinki, Finland

## Abstract

Psoriatic skin differs distinctly from normal skin by its thickened epidermis. Most gene expression comparisons utilize full-thickness biopsies, with substantial amount of dermis. We assayed the transcriptomes of normal, lesional, and non-lesional psoriatic epidermis, sampled as split-thickness skin grafts, with 5′-end RNA sequencing. We found that psoriatic epidermis contains more mRNA per total RNA than controls, and took this into account in the bioinformatic analysis. The approach highlighted innate immunity-related pathways in psoriasis, including NOD-like receptor (NLR) signaling and inflammasome activation. We demonstrated that the NLR signaling genes *NOD2*, *PYCARD*, *CARD6*, and *IFI16* are upregulated in psoriatic epidermis, and strengthened these findings by protein expression. Interestingly, PYCARD, the key component of the inflammasome, showed an altered expression pattern in the lesional epidermis. The profiling of non-lesional skin highlighted *PSORS4* and mitochondrially encoded transcripts, suggesting that their gene expression is altered already before the development of lesions. Our data suggest that all components needed for the active inflammasome are present in the keratinocytes of psoriatic skin. The characterization of inflammasome pathways provides further opportunities for therapy. Complementing previous transcriptome studies, our approach gives deeper insight into the gene regulation in psoriatic epidermis.

Psoriatic skin is characterized by the hyperproliferation and abnormal differentiation of keratinocytes and infiltration of inflammatory cells. Components of the cornified envelope, the outermost layer of epidermis, are prematurely synthesized in the spinous layer that is thicker and disorganized in psoriasis. The inflammatory infiltrate consists mainly of dendritic cells, macrophages, and T cells in the dermis and neutrophils with some T cells in the epidermis^1^,^2^. Gene expression in the epidermis is dramatically altered during the pathogenesis of psoriasis. For example, several genes of the epidermal differentiation complex (EDC) region (1q21) are upregulated in the psoriatic lesions. These include genes that play a role in the generation and maintenance of the epidermis: cornified envelope precursors (e.g., small proline-rich proteins; SPRRs), late cornified envelope proteins (LCEs), and signaling proteins (e.g., sS100 calcium-binding proteins; S100s). The EDC region also contains the psoriasis susceptibility locus 4 (*PSORS4*)^3^,^4^.

The regulation of inflammation in the psoriatic skin requires cross-talk between the keratinocytes and the immune cells. Keratinocytes produce several anti-microbial peptides and proteins (e.g., LL37, β-defensis, and interferon-γ) that attract immune cells and shape their functions. They also respond to immune cell-derived cytokines, such as interferons, interleukin-17, and the interleukin-20 family of cytokines, and in turn produce proinflammatory cytokines (interleukin-1 and TNF-α)^1^. In addition, they recognize pathogens and endogenous cellular stress signals via pattern recognition receptors (PRRs): e.g., NOD-like (NLR) and RIG-like receptors (RLR), and therefore mediate immune responses^5^.

Several microarray studies of the psoriatic lesional skin have revealed a large number of differentially expressed genes (DEGs) in comparisons with control and non-lesional skin^6^,^7^. Recently, RNA sequencing (RNAseq) has provided a new alternative to microarrays; so far, two RNAseq studies on psoriasis have been published^8^,^9^. However, most of the previous psoriasis transcriptome analyses used full-thickness skin samples. Here we aimed to focus on epidermal changes in psoriasis. We utilized RNA 5′-end targeted sequencing for split-thickness skin grafts (SG) that are composed of the epidermis (Supplementary Figure S1) and include much less dermis than the full-thickness samples. We collected samples from the psoriatic lesional and non-lesional skin from patients and healthy skin from controls. We also employed data normalization with synthetic spike-in RNA, which enables more accurate comparison of gene expression in heterogeneous samples where total mRNA levels per cell may be highly different^10^,^11^,^12^. Our results show the power of RNAseq over the microarray studies, providing a more comprehensive view of altered signaling pathways both in non-lesional and lesional psoriatic skin. The sensitivity of the RNAseq method, together with the skin graft samples, allows a more in-depth identification of altered components in each pathway, making it possible to get a better overall understanding about affected pathways and networks in psoriatic epidermis.

## Results

### RNAseq of psoriatic skin grafts identified a substantial number of differentially expressed transcripts

Total RNA samples of 10 ng, extracted from nine control (C), five psoriatic non-lesional (PN), and six lesional (PL) SGs (Table 1, Supplementary Table S1), were subjected to 5′-end RNAseq^10^. For differential expression analysis, we employed the SAMstrt statistical package, which is based on synthetic spike-in RNA normalization of data^11^,^12^.

**Figure 1:**
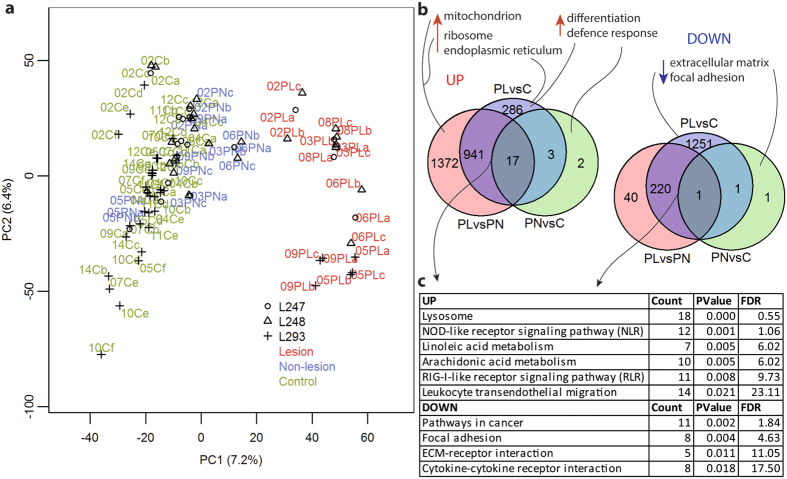
Expression profiling of lesional and non-lesional psoriatic and control skin by RNAseq. (**a**) Principal component analysis demonstrates clustering of the spike-in normalized sample groups PL (red), PN (blue), and C (green). Percentages are contribution ratios. The three libraries have different symbols; L247 and L248 indicate Run1 and L293 Run2 (description in Supplementary Table S1) (**b**) Venn diagram of DEGs from the three comparisons: PLvsPN, PLvsC, and PNvsC (FC > 1.5 or < 0.75, FDR < 0.05). The most significant GO categories are shown (red arrow indicates up- and blue downregulated). (**c**) Enriched KEGG pathways of DEGs that are common to the PLvsC and PLvsPN comparisons (FDR < 0.25).

We estimated the dissimilarity between the samples by principal component analysis (PCA) (Fig. 1a), which illustrates significant overlap of the healthy skin samples (PN and C) but separation from the lesional samples. Some non-lesional samples clustered between the control and lesional samples, suggesting changes already in non-lesional skin.

The group-wise (GW) comparisons of transcripts between lesion and control (PLvsC) or non-lesion (PLvsPN) revealed 2436 and 3541 upregulated and 2550 and 494 downregulated differentially expressed transcripts (DETs), respectively (Supplementary Table S2a–f) (Fold Change (FC) > 1.5 and <0.75, False Discovery Rate (FDR) < 0.05). Positional analysis with Gene Set Enrichment Analysis (GSEA)^13^ revealed, in both comparisons, the enrichment of upregulated transcripts from *PSORS4* locus (Supplementary Table S3). GW comparison of non-lesion with control skin (PNvsC) revealed 35 DETs (Supplementary Table S2). Of the DETs from all comparisons, we selected classes =, c, j, e, and o (71% of all classes, Supplementary Table S2) to represent the genes (DEGs). We identified 2720, 2610, and 25 DEGs from the PLvsC, PLvsPN, and PNvsC comparisons, respectively (Fig. 1b). We also compared gene expression pair-wisely (PW) between lesional and non-lesional skin from each psoriatic patient separately (data not shown), and analyzed the DEGs that were common to all patients.

### Expression profiling of lesional psoriatic skin highlighted functions involved in epidermal homeostasis

**Figure 2:**
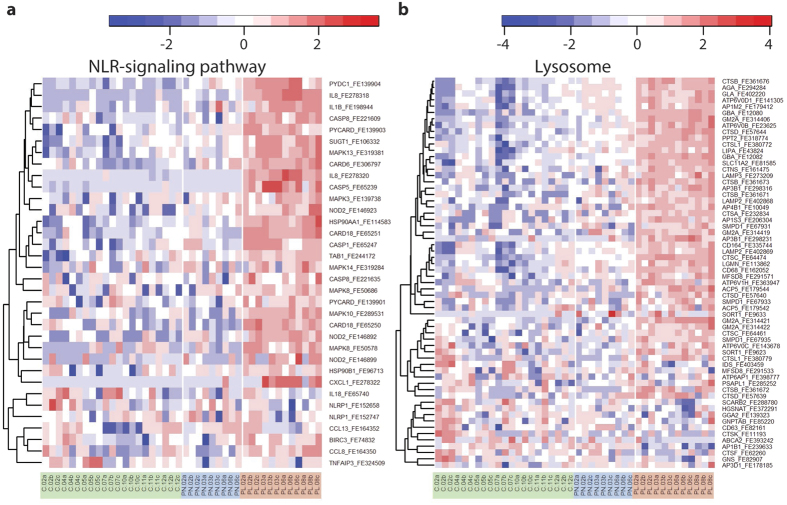
Heat maps of NLR signaling and lysosome pathways. Gene expression in PL, PN, and C samples (Run1). Color key: red represents upregulated and blue downregulated expression (row Z-score). Run2 heat maps are shown in Supplementary Figure S2.

Using the Database for Annotation, Visualization and Integrated Discovery (DAVID)^14^ and WebGestalt^15^ tools, we analyzed the DEGs from the PLvsPN and PLvsC comparisons (GW). The comparisons gave similar results (Supplementary Table S4). Therefore, we analyzed the DEGs that are shared in these two comparisons (the intersection of PLvsPN and PLvsC, Fig. 1b). The functional annotation analysis of upregulated genes from the intersection highlighted gene ontology (GO) groups related to *epidermal differentiation*, including the *LCE* and *SPRR* genes. Functions, such as *defense response*, *oxidoreductase*, *protease*, and *lipid degradation* were among the most significant clusters as well. The analyses also identified *CARD* (*CAspase Recruitment Domain*) and *caspase* gene families. The most highly upregulated genes of lesional samples were enriched especially in *defense response* and *hydrolase* (data not shown). KEGG pathway analyses identified e.g., *lysosome*, *NLR*, and *RLR signaling* (Figs 1c and 2, Supplementary Figure S2a,b).

The analysis of the upregulated genes from the GW-PLvsPN comparison highlighted GOs related to *mitochondria* and *oxidative phosphorylation* that showed enrichment in the PW comparison as well, but were not among the most significant and largest groups in the GW-PLvsC comparison. This might result from the heterogeneity of the patients (Table 1). We analyzed the upregulated genes of PLvsPN by GSEA as well (Supplementary Table S4k). These results are consistent with the DAVID and WebGestalt analyses.

The intersection of the GW-PLvsPN and -PLvsC (Fig. 1b) contained 220 downregulated genes that were enriched in such functions as the *extracellular matrix*, *blood vessel development*, and *cell junction*, which may also be a reflection of the lower proportion of dermis in the PL samples, instead of real downregulation of the genes. The pathway analysis recognized *pathways in cancer* (Supplementary Figure S1c), *cytokine-cytokine receptor interaction*, and *focal adhesion* as well. Separate analyses of the downregulated genes from PLvsPN and PLvsC gave rather similar results with the intersection of these two comparisons (Supplementary Table S4a–g). However, PLvsC recognized many more DEGs than PLvsPN (1473 and 261, respectively); the enrichment analyses thus revealed pathways that were not recognizable in the PLvsPN comparison, such as *Wnt*, *TGF-beta*, and *Notch signaling* that all have been identified in previous studies on psoriasis^16^.

### Pathways related to innate immunity were highlighted in psoriatic lesional skin

NLR signaling was highlighted in all the comparisons with lesional samples (GW-PLvsC, GW-PLvsPN, and PW-PLvsPN) (Figs 1c and 2a, Supplementary Figure S2a). RLR signaling and cytosolic DNA sensing pathways rose up as well. All three pathways shared several components (Table 2). The DEGs (PLvsPN) belonging to the NLR signaling included highly upregulated transcripts: *NOD2*, *CARD6*, *CARD18*, *CASP5*, *IL1B*, *IL8*, and *CXCL1* (FC > 1 × 10^8^). Also several other components involved in the NLR signaling were identifiable, such as *NLRP10*, *NLRX1*, *CASP1*, *CASP8*, and *PYCARD* (ASC). Most of these genes were identifiable in the PLvsC comparison as well. Furthermore, the receptors of the cytosolic DNA sensing and RLR pathways; DNA-binding receptors *AIM2* and *IFI16* and RNA helicase proteins *IFIH1* and *DDX58* (*RIG-I*), were induced in the lesional samples. Also several other RLR-related transcripts were upregulated, including *ISG15* and *CYLD* (Table 2).

**Figure 3:**
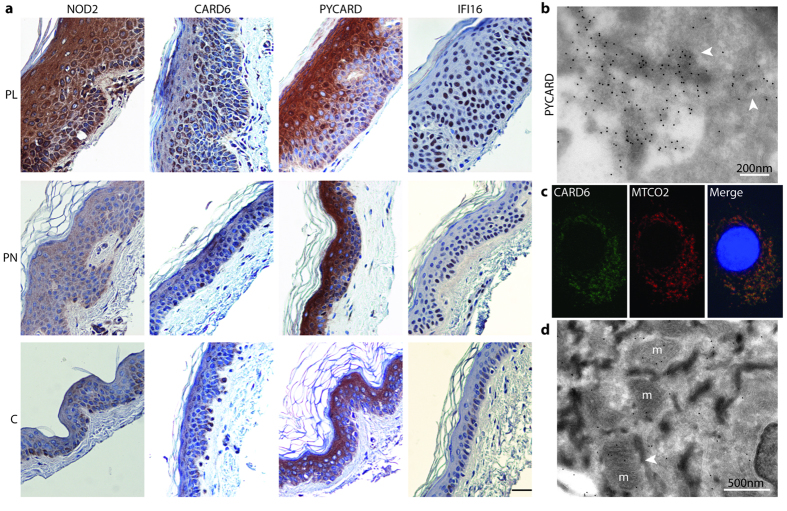
Induced expression of NOD2, CARD6, PYCARD, and IFI16 in psoriasis lesions. (**a**) Immunohistochemistry shows stronger NOD2, CARD6, PYCARD, and IFI16 staining in PL (upper) than in controls (lower). PN (middle) and C are almost negative for NOD2 and CARD6. IFI16 is predominantly in nuclei of PL and PN. In controls IFI16 expression is weak and cytoplasmic. PYCARD is strongly induced in cytoplasm but also some nuclei are positive in PL and PN. Controls exhibit only a few PYCARD positive nuclei. Scale bar 50 μm. (**b**) IEM of PL shows PYCARD clusters in cytoplasm. (**c**) Immunofluorescence of keratinocytes colocalizes CARD6 with a mitochondrial MTCO2. (**d**) IEM of psoriatioc lesional samples also localized CARD6 in the mitochondria (arrow head).

We showed recently, by qPCR, that our RNAseq protocol enables the accurate quantitation of gene expression in skin^12^. Patient samples of the present study were processed and analyzed at the same time as samples of the previous study Katayama *et al.* Here, we validated the upregulation of *CARD6*, *IFI16*, *PYCARD*, and *IL8* in lesional skin samples by qPCR (Supplementary Figures S3 and S4a). We also used immunohistochemistry to examine and verify the expression and localization of the proteins encoded by the differentially expressed genes *NOD2*, *PYCARD*, *IFI16*, *CARD6*, and *NLRP10* (Fig. 3a, Supplementary Figure S4). We selected these proteins in particular because their expression pattern has not been thoroughly studied in psoriatic skin before, or it has remained unclear. Our antibody staining demonstrated that in most lesional samples; NOD2 expression was induced in the epidermis, including keratinocytes, when compared with the non-lesional or control samples. In the psoriasis non-lesional and lesional skin: expression varied between individuals (Supplementary Figure S4). In the non-lesional samples, especially, there was more variation from weak to increased expression. On the cellular level, NOD2 was expressed in cytoplasm and on the cell membrane in some cells. Epidermal PYCARD staining was observed in all sample groups. The expression level and pattern, however, differed between the sample groups (Fig. 3a): in lesions the expression was strongly induced in cytoplasm, and in some cells in nuclei. The control skin exhibited only a few PYCARD positive nuclei, and its overall staining was weaker than in the lesions. In the non-lesional skin, the overall PYCARD staining was stronger than in controls and some samples showed nuclear staining. The cytoplasmic PYCARD induction of lesional samples was observed also in immuno electron microscopy (IEM) (Fig. 3b, Supplementary Figure S4). Interestingly, in some keratinocytes the gold labeling formed clusters (diameter around 500 nm) that localized with the cytoplasmic membrane structures, possibly small vesicles. The IFI16 staining was weak in most controls, whereas strongly upregulated in lesional epidermis and localized into cell nuclei (Fig. 3a). In some controls, we detected a weak cytoplasmic IFI16 expression and only a few positive nuclei. In contrast, in the lesional and non-lesional samples; cytoplasmic expression was hardly detectable. In the non-lesional samples, the expression varied from weak to strong and was localized to nuclei. We found strong cytoplasmic CARD6 expression in keratinocytes of lesional samples (Supplementary Figure S4). CARD6 was detectable also in nuclei, and as a granular cytoplasmic staining, possibly representing mitochondria, as shown by immunofluorescent staining of cultured keratinocytes (Fig. 3c). The control skins were almost CARD6 negative (Fig. 3a). The non-lesional samples resembled controls but some showed induced CARD6 expression in epidermis. The IEM of lesional skin confirmed the mitochondrial localization in keratinocytes (Fig. 3c) and revealed CARD6 expression in cell-cell contacts as well (Supplementary Figure S4). We found a pronounced cytoplasmic NLRP10 staining all over the epidermis, and could not observe any difference between psoriatics and controls (Supplementary Figure S4e). Our staining result agreed with previous findings in normal skin^17^.

### RNAseq of skin graft samples refined previous findings in psoriasis

**Figure 4:**
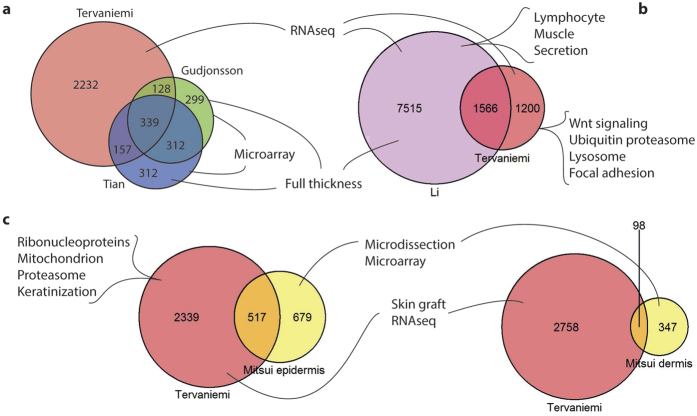
Comparison of our RNAseq data with other transcriptomics analyses of psoriatic skin. Venn diagrams of RNAseq from our SGs (either PLvsC or PLvsPN) (Tervaniemi) in comparison with **(a)** micro arrays (PLvsPN) (Gudjonsson and Tian) and **(b)** RNAseq (PLvsC) of full-thickness samples (Li) and **(c)** micro array of microdissection samples (PLvsPN) (Mitsui). The unique genes of each assay were enriched in the pathways (Li) or GOs (Mitsui) presented, although similar pathways were identifiable in the different studies.

We compared our RNAseq data of SG samples with previous microarray and RNAseq studies. First we compared our data with two large microarray studies by Gudjonsson *et al.* and Tian *et al.*^6^,^7^ (Fig. 4a; Supplementary Table S5), conducted with full-thickness skin samples of the psoriatic lesional and non-lesional skin, and detected 2232 DEGs that were identifiable only in our study. The recent RNAseq by Li and colleagues^9^ differs from our study in several ways including sample number and type, sequencing, and normalization methods. Instead of SGs, they used the full-thickness biopsies of lesional and control skin. Similar functional categories and pathways were identifiable in both RNAseq studies. The shared part included 1566 DEGs, but numerous unique transcripts were recognized as well; 1200 and 7515 DEGs in the SG and full-thickness skin, respectively (Fig. 4b, Supplementary Table S5). Genes that were unique for SGs were enriched in such categories as *Wnt signaling* (e.g., *PPR2R1B*, *PPP2CA*, *APC*), *ubiquitin proteasome pathway*, *lysosome* (e.g., V-ATPases, *CTSA, CTSD*), and *focal adhesion* (e.g., *IGF1R*, *ITGA2*, *COL5A3*) (Supplementary Table S5c). In NLR signaling we identified DEGs that were not recognized in the full-thickness samples with the FC ≥ 1.5 (Table 2; e.g., *CASP1*, *CASP8*, *CARD18*, *CYLD*, and *TNFAIP3*). GOs related to *lymphocyte* (upregulated), *muscle*, or *secretion* (downregulated) were among the top enrichment groups in the full-thickness samples^9^ whereas in SGs they were missing or not among the significantly altered ones (Supplementary Table S5). This may result from the different proportions of dermis in the SG and full-thickness samples.

In a recent microarray study; Mitsui *et al.* examined gene expression in epidermal and dermal samples collected from the lesional and non-lesional skin by microdissection^18^. When we compared our DEGs with their epidermal data, 517 were common and the number of unique DEGs in SGs was 2339 and 679 in microdissected epidermis (Fig. 4c, Supplementary Table S5d,f). Our RNAseq identified, for example, several *LCE*, *SPRR*, and *KRT* genes that were undetectable in the microarray assay. Among our unique transcripts we identified 13 DEGs belonging to the NLR signalling, these including genes such as *CARD6*, *CARD18*, *CASP8*, *IL1B*, and *PYDC1*. Transcripts for *NOD2*, *PYCARD*, *DDX58*, *CASP1*, and *IL8* were upregulated in both studies (Table 2). Only 3% of our DEGs were detectable in their dermal fraction, in agreement with the amount of dermis in our SGs (Supplementary Table S5e).

We aimed at minimizing the amount of dermis when collecting the SGs. With our dermatome technique, however, the proportion of dermal compartment varied between the samples and still remained higher in the PN and control than in the PL samples (Fig. 3, Supplementary Figure S1). We checked whether the variation of the amount of dermis between the skin samples has an effect on our RNAseq results, by comparing the expression of fibroblast specific genes, *COL3A1* and *COL1A2*, between the three sample groups (Supplementary Figure S5). We could see a slight decrease in the expression of these markers in some of the samples. As we have much less dermis in our samples than there had been in the full-thickness samples, the relative decrease of the dermal proportion in the lesional samples, when compared with the non-lesional ones, is more pronounced in SGs than in full-thickness samples. The architecture and thickening of the epidermis might create some downregulation of the dermal components as well. The number of downregulated DEGs in the lesional SG samples, however, is lower than the number observed in the full-thickness studies.

### Expression profiling of non-lesional skin showed upregulation of *PSORS4* and mitochondrially encoded transcripts

**Figure 5:**
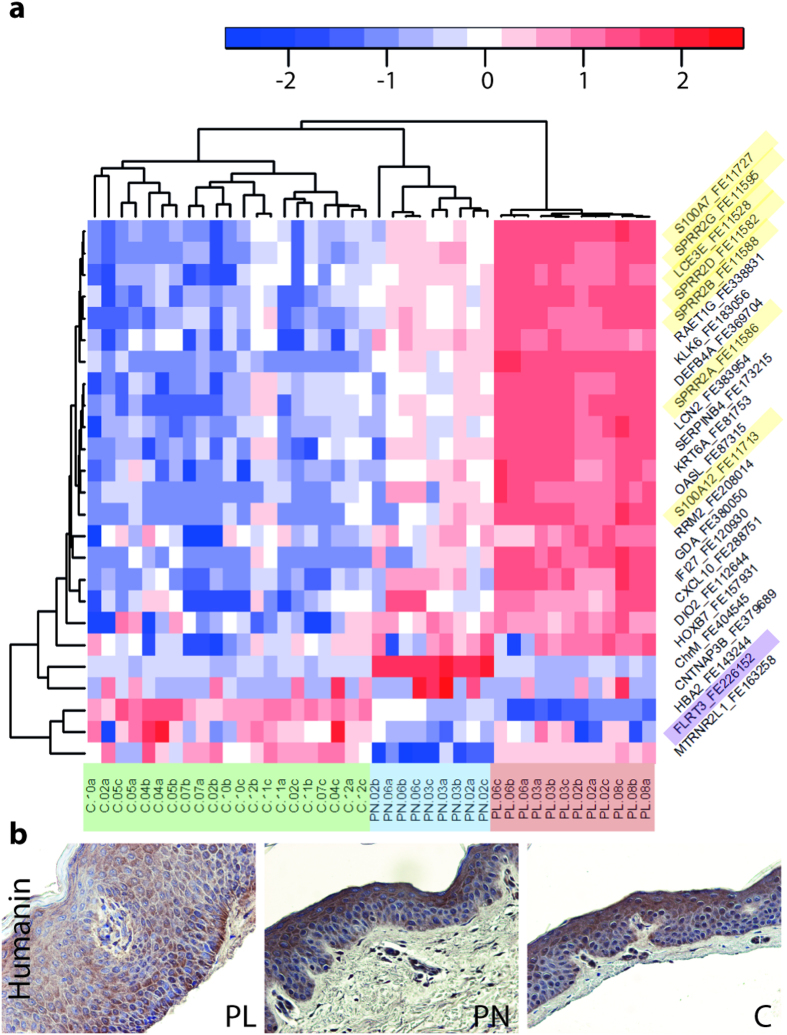
Gene expression of non-lesional psoriatic skin. (**a**) Heat map of DEGs from the PNvsC comparison; gene expressions in samples (PL, PN, and C) of the Run1. Gene name includes class (DEGs:o/e/c) and first exon codes. Color key: red represents upregulated and blue downregulated expression according to the color intensity (row Z-score). Most of the induced transcripts (PNvsC) are highly upregulated in PL (PLvsC), as *PSORS4* transcripts (yellow). *PSORS12* (purple) transcript *FLRT3* is downregulated in PN. The most highly upregulated transcript in PNs, ChrM_o_FE404545, represents *MTRNR2*, which shows no induction in PL. Heat map of the Run2 is shown in Supplementary Figure S6. (**b**) Immunostaining with the MTRNR2 (humanin) antibody reveals expression in PL, PN, and C skin.

Comparison of non-lesional skin with control showed that 35 transcripts were differentially expressed (Supplementary Table S2a and b): 28 were upregulated (FC > 1.5) and 7 downregulated (FC < 0.75). Interestingly, 12 of the transcripts mapped to the known *PSORS* loci; *PSORS4* was the most represented among the upregulated transcripts (Supplementary Table S3b) as shown also in previous assays. The upregulated genes (Fig. 5a, Supplementary Figure S6) were enriched especially for *keratinocyte* and *epidermis differentiation* but also for *defense response* (Fig. 1b; Supplementary Table S4c). Most of the upregulated transcripts (PNvsC) were induced also in the lesions (PLvsC), except *CNTNAP3B* and the mitochondrial transcripts (ChrM) named in the alignment step as *TVAS5*, both of which have not been implicated in psoriasis before (Fig. 5a, Supplementary Figure S6). The most frequent *TVAS5* reads map at the start site of mitochondrially encoded 16S ribosomal RNA (*MTRNR2* gene) (Supplementary Figure S6b,c) that also encodes for an antiapoptotic polypeptide called humanin.

Among the downregulated genes (PNvsC), we identified only three DEGs (Table S2b) one of which was the nuclear gene homolog of *MTRNR2*, namely *MTRNR2L1* (humanin-like). Because of the high sequence similarity between humanin-like genes^19^, the specific quantitation of humanin and its derivate by qPCR was difficult. We demonstrated by immunohistochemistry (Fig. 5b, Supplementary Figure S6e) that humanin and humanin-like proteins are strongly expressed in keratinocytes in all the three sample types but we were unable to detect any difference between the psoriatic and healthy skin. Based on our RNAseq data, however, the gene expression of the peptides is dysregulated in the non-lesional skin, suggesting that the regulation of apoptosis might be disturbed already before the lesions develop. It remains to be studied whether humanin and its derivates play a role in the pathogenesis of psoriasis.

## Discussion

Many studies are available that compare the transcriptomes of psoriatic and healthy skin using full-thickness biopsies. Our approach is different from such studies in two aspects: we focus on the epidermis and use a bioinformatics approach that takes into account the systematically higher amount of mRNA per total RNA in psoriatic skin. Our results highlight functions related to epidermal homeostasis in lesional skin. The most obvious difference between full-thickness and SG transcriptomes of psoriasis was that *lymphocyte*, *muscle contraction*, and *secretion* were not highlighted in SGs. The difference likely results from the large amount of dermis in full-thickness biopsies, especially as the latter two gene sets are enriched among the downregulated genes in lesions. Recently, it has been suggested that dermis-derived transcripts are driven downward by the expansion of epidermis in psoriatic lesions when compared with healthy skin^9^. Similar effect was also observable in the SG samples; the relative proportion of dermis is higher in the healthy skin samples than in the lesional samples, even though the amount of dermis is much lower in SGs than full-thickness samples. Another reason for differences in the transcriptome results might be the gene length bias^20^, which we avoid with the use of the 5′-end targeted RNAseq method. We conclude that the advantage of skin graft samples is to get results that highlight changes in epidermal gene expression and thus improve resolution of the expression analysis.

**Figure 6:**
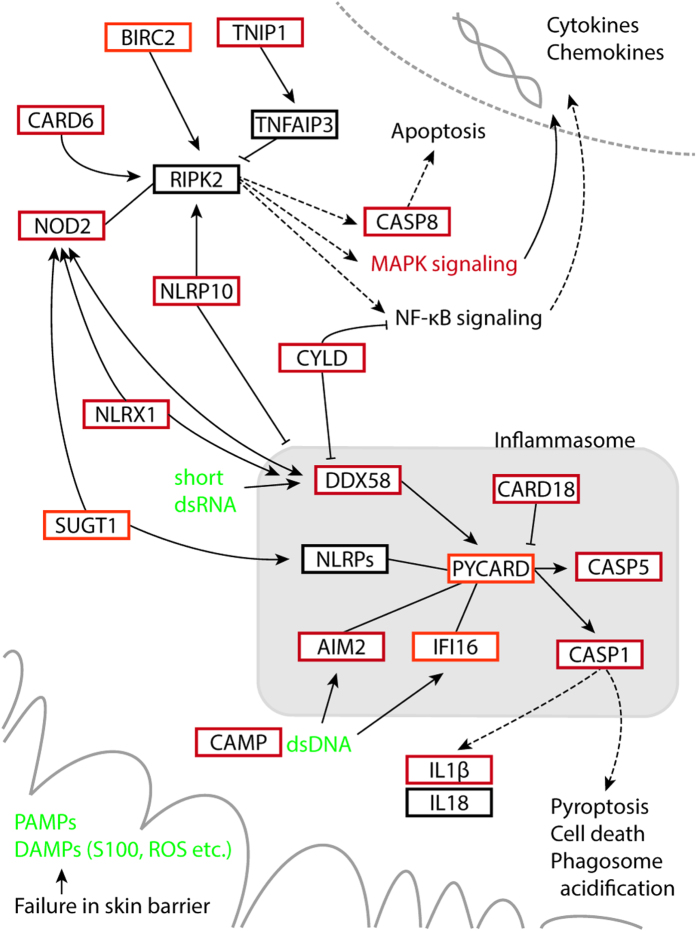
Model of NLR signaling in keratinocytes of psoriatic skin. Exposure to DAMPs and PAMPs (damage- and pathogen-associated molecular patterns) triggers activation of NLR signaling and inflammasome. NOD2 operates via RIPK2 by activating the NF-kB and MAPKs pathways, leading to production of inflammatory mediators. The NLR signaling is also linked to apoptosis and inflammasome activation. The latter regulates CASP1; required for the maturation of IL1B and IL18. Inflammasome consists of the NLR protein, CASP1, CASP5, and the adaptor protein PYCARD. Also the DNA-binding receptors AIM2 and IFI16, and the RNA-sensing protein DDX58, operate via inflammasome. Cytosolic DNA triggers AIM2 and IFI16 inflammasome, whereas anti-inflammatory CAMP inhibits their function. DEGs upregulated in PLvsPN or in both PLvsC and PLvsPN are shown as orange and red, respectively.

We identified more DEGs for each pathway than previous microarray studies. Recognition of the NLR signaling (Fig. 6), including inflammasome activation, is a good example of the power of RNAseq. Of the dysregulated genes involved in the NLR signaling, we studied *NOD2*, *PYCARD*, *IFI16*, *CARD6*, and *NLRP10*. Their induction in lesional skin is identifiable in previous transcriptome studies^9^,^18^ (Table 2), except for *NLRP10*. Here, we demonstrated their protein expression in psoriatic epidermis, by using immunohistochemistry. We showed that NOD2 expression was induced in the psoriatic epidermis, especially in keratinocytes. The risk alleles of NOD2 have been linked to several inflammatory diseases, including atopic eczema and arthritis^21^, but their role in psoriasis is still questionable^22^. NLRs, such as our DEGs *NOD2*, *NLRP10*, and *NLRX1*, operate via *RIPK2* by modulating the nuclear factor-kB and mitogen-activated protein kinase pathways that lead to the production of chemokines, cytokines, and antimicrobial peptides^21^,^23^. *CARD6* has not been linked to psoriasis previously though it was highly upregulated in our PL. Its function is unclear; it may regulate NOD2-RIPK2 signaling^24^. Based on our immunofluorescence and IEM staining, CARD6 is localized both in the cytoplasm and the mitochondria. Interestingly, a small fraction of NOD2 is also associated with mitochondria^25^.

We identified also several other genes (Fig. 6) that are linked to the inflammasomes, which are infection or stress-activated cytoplasmic protein complexes. They regulate CASP1 activity, which is required for the processing and maturation of inflammatory cytokines IL-1 and IL-18. They consist of the NLR receptor protein, CASP1, sometimes also CASP5, and the adaptor protein PYCARD that is a key component of the inflammasomes. The inflammasome assembly is trigged by the interaction of PYCARD with the receptor molecule. Using its CARD domain, PYCARD brings pro-CASP1 into close proximity, which initiates CASP1 self- cleavage and the formation of the active tetrameric CASP1. Upregulation of *PYCARD* and *CASP1* in lesional skin may promote the formation of large multiprotein complexes consisting of multimers of PYCARD dimers and several CASP1 molecules. The exact composition of inflammasome depends on the activator that initiates the assembly of the inflammasome; receptors are specific for individual activators^23^. Here, we showed that PYCARD was predominantly cytoplasmic in lesional epidermis, but in some cells also nuclear. Whereas the control skin exhibited only a few positive nuclei, and the overall expression was lower than in psoriasis. In keratinocytes of lesional skin our IEM detected cytoplasmic PYCARD clusters that may represent inflammasomes.

The genes for the most studied inflammasome receptors, NLRP1 and NLRP3, were not among our DEGs, but they have been associated with psoriasis^26^,^27^. We demonstrated that the NLRP10 protein is strongly expressed in healthy as well as in psoriatic skin. It may inhibit the inflammasome assembly^28^ and there is a polymorphism in *NLRP10* that is associated with atopic dermatitis, but not with psoriasis^21^,^29^.

The DNA-sensing receptors encoded by *AIM2* and *IFI16*, which were upregulated in the lesional samples, form an inflammasome with PYCARD (Fig. 4)^23^,^30^,^31^. They are also implicated in psoriasis^32^,^33^,^34^. Intriguingly, there is abundant cytoplasmic DNA in keratinocytes of psoriatic lesions, and thus DNA-sensing receptors may play a role in the pathogenesis of psoriasis^32^,^35^. AIM2 is among the most highly upregulated PRRs in lesional skin^33^. Here, we showed that IFI16 was also strongly increased in the lesions, and predominantly present in nuclei. This agrees with previous findings; IFI16^31^ possibly activates PYCARD already in the cell nucleus^30^. It has been suggested that in psoriasis lesions, in a subpopulation of keratinocytes, IFI16 translocates from cell nuclei into the cytoplasm, whereas in non-lesional skin it stays in nuclei^34^. Our immunostaining, however, did not support the translocation of IFI16. The most obvious difference was strong nuclear staining in lesions, whereas weak expression in controls.

RNA-sensing receptors, *IFIH1* and *DDX58* (*RIGI*), which were induced in the lesional samples, are well known susceptibility genes in psoriasis^36^,^37^. DDX58 operates via inflammasome and there is interplay between the RLR and NLR pathways; e.g., DDX58 and NOD2 regulate each other (Fig. 4)^38^. *CYLD*, a psoriasis candidate gene^39^ and a DEG identified by us, acts in both pathways^40^,^41^.

As a summary; we among others have observed that inflammasome and innate immune receptors are upregulated in keratinocytes of psoriatic skin^36^,^42^,^43^. Inflammasome activation, however, is regulated at several levels^23^ and expression alone does not automatically mean a biological relevance. It is known that normal human keratinocytes constitutively express inflammasome proteins; for instance innate immune receptors are constantly monitoring for signs of infection, cellular damage, or stress factors. Based on our results; AIM2 and IFI16 are highly expressed inflammasome receptors in psoriasis, whereas NLRP3 was not among the DEGs. Recently it was suggested that the basal expression of NLRP3 is not sufficient for the inflammasome activation in resting cells. Instead, the NLRP3 expression is transcriptionally induced at first, and only after posttranscriptional modifications, its inflammasome assembly is activated^23^. Post-translational modifications, such as phosphorylation, ubiquitination, and even proteolysis, are necessary for the activation of certain inflammasome receptors. The subcellular location and trafficking of inflammasomal components are also important for the regulation of their activity. Therefore, alterations in the expression pattern or subcellular location that we observed with PYCARD and IFI16, may suggest that certain types of complexes may form in psoriatic skin. The exact composition and activation mechanisms of inflammasomes in psoriasis remain to be determined.

In conclusion, RNAseq with absolute rather than relative RNA quantification, combined with the use of skin graft samples, allowed an improved recognition of the altered signaling pathways in psoriasis. Compared with the previous RNAseq-based psoriasis studies on the full-thickness skin samples, our approach provided more information about the transcriptional dysregulation in the epidermis. A good example is the recognition of the NOD-like receptor signaling and functions related to it, such as inflammasome activation in keratinocytes. The 5′-end RNAseq method allowed the precise determination of transcription start sites as well; it remains to be studied whether aberrant gene expression patterns that promote the pathogenesis of psoriasis arise from alternative promoter usage.

## Methods

### Sample collection, RNA extraction, and RNAseq

The Institutional Review Board of the Helsinki University Central Hospital approved the collection of skin samples. All subjects involved in this study gave written informed consent and the study followed the Declaration of Helsinki Guidelines. Split-thickness skin grafts and primary keratinocytes were harvested and processed as previously outlined^12^,^44^,^45^ and the RNAseq of control samples was recently described elsewhere^12^. Briefly, we collected lesional and non-lesional samples from six psoriatic patients and normal healthy skin from nine controls (Table 1). Absolute washout period was not required. Psoriatic skin samples PL and PN were all from the same location, from buttock. Location of control samples varied, as samples were collected from breast reduction and microvascular free flap surgery patients. All control samples, however, were from the areas that are normally protected from sun. Split-thickness skin graft (SG) samples were harvested by a compressed air-driven dermatome (Zimmer^®^, Warsaw, IN) by using a fixed setting for thickness (4–6/1000 inches) to obtain samples including the full epidermis. We aimed at getting minimal dermis involvement (Supplementary Figure S1). However, some dermis remained in the control and PN samples, whereas in the PL samples the amount of dermis was minimal. Skin specimen used for RNAseq and immunohistochemistry were taken from the same larger SG sample, and before the RNA isolation the quality of SG samples was examined from haematoxylin-eosin (HE)-stained paraffin sections. Skin sections shown in immunohistochemistry panels in Figs 3 and 5, and Supplementary Figure S1 represent the skin samples used for the RNA isolation. Total RNA was extracted by miRNeasy kit (Qiagen) from the skin samples and its quality was controlled with Bioanalyzer (RIN for all samples > 8). STRT libraries of PL and PN samples were prepared and sequenced at the same time as controls^12^. The samples were prepared into three different libraries, as demonstrated in Supplementary Table S1. Total RNA samples (three replicates for each sample, 10 ng of each replicate) were used for RNA sequencing library preparation according to the STRT protocol^10^, which was adjusted for 10 ng samples. The libraries were sequenced using an Illumina HiSeq 2000 instrument. Redundancy was reduced according to UMI^46^, and the non-redundant reads were demultiplexed and trimmed by demlt tool in ruby-bio-gadget (https://github.com/shka/ruby-bio-gadget). The demultiplexed reads were aligned to hg19 human reference genome, ArrayControl RNA spikes and human ribosomal DNA complete repeating unit [GenBank: U13369] by TopHat^47^. The aligned STRT reads were assembled by sample types using Cufflinks^48^, and 5′-end regions of the assembled transcripts were merged as Transcript Far 5′-ends (TFEs). TFEs were compared by Cufflinks with UCSC genes to annotate. Reads aligned within the TFEs were counted by samples again, and normalized by the spike-in based normalization^11^. Differential expression analysis was performed by SAMstrt^11^,^12^. In RNAseq data; extremely high FC values such as 1 × 10^8^ results from the calculation method used in SAMstrt. In the comparison between zero transcripts and some transcripts to avoid calculation errors by zero division, SAMstrt adds small random numbers to all normalized transcript counts and then calculates the FC. We performed PCA with the scaling but non-centering preprocess steps. Correlation of gene expression with PC was estimated by SAMstrt quantitative response test. Scores of samples on a PC were given as the quantitative values, and threshold of the significantly correlated gene is Local-FDR < 1%.

### Quantitative PCR

cDNA synthesis was carried out using random primers and SuperScript III First-Strand synthesis system for RT-PCR (18080-151, Invitrogen) according to manufacturer’s instructions. 10–20 ng of cDNA (RNA) was applied to each qPCR assay and each sample was run in three replicates. qPCR was carried out using an ABI PRISM 7900HT Sequence Detection System with Fast SYBR^®^ Green Master mix (4385617, both from Applied Biosystems) according to manufacturer’s instructions. The primer sequences for *PYCARD*, *CARD6*, *IFI16*, and *IL8* are shown in Supplementary Figure S3a. *RPL13* and *GAPDH* were used as reference genes for normalization.

### Immunohistochemistry

Formalin fixed paraffin sections (5 um) were stained with different anti-human antibodies using the peroxidase-based ImmPRESS™ Reagent kit (Vector MP-7500) and ImmPACTTMDAB™ peroxidase substrate kit (Vector SK-4105). Epitope retrieval was carried out by a heat-mediated method in sodium citrate buffer pH 6.0 for 20 minutes. The primary antibodies were: ASC (AdipoGen, AG-25B-0006-C100), TMS1 (Proteintech, 10500-1-AP), CARD6 (Novus Biologicals, NBP2-15704), IFI16 (Novus Biologicals, NBP1-83118), IFI16 (Abcam, ab55328), NLRP10 (Novus Biologicals, NBP1-85556), and Humanin (Thermo Fisher, PA1-41325). Normal rabbit IgG was used as a negative control (Dako, X0903).

### Immunofluorescence

For immunofluorescence studies, we cultured cells extracted from full-thickness samples^12^. The cells were grown on cover slips with Rat Tail Collagen I (Gibco, Invitrogen) coating, and fixed with methanol for 5 min at −20 °C^56^. The cover slip samples were incubated one hour at room temperature with the MTCO2 (Abcam, ab3298) and CARD6 antibodies. Alexa Fluor 555 and 488 conjugated IgGs (Invitrogen, Molecular Probes) were used as secondary antibodies and the nuclei stained with DAPI (Sigma-Aldrich). The pictures were taken with Zeiss LSM 5 Duo confocal microscope.

### Immunoelectron microscopy

Skin biopsies were fixed with 4% paraformaldehyde-PBS solution for 6 to 12 h followed by immersion in 2.3 M sucrose-PBS solution. Samples were frozen in liquid nitrogen, and thin cryosections were cut with a Leica Ultracut UCT microtome. Sections were first incubated in 0.1% glycine-PBS, then in 1% BSA, labeled with antibodies against PYCARD or CARD6 followed by incubation with protein-A gold conjugate. Labeling was detected with a Tecnai G2 Spirit transmission electron microscope (FEI Company) and Quemesa CCD camera (MSIS GmbH).

## Additional Information

**How to cite this article**: Tervaniemi, M. H. *et al.* NOD-like receptor signaling and inflammasome-related pathways are highlighted in psoriatic epidermis. *Sci. Rep.* **6**, 22745; doi: 10.1038/srep22745 (2016).

## Supplementary Material

Supplementary Information

Supplementary dataset 1

Supplementary dataset 2

Supplementary dataset 3

Supplementary dataset 4

Supplementary dataset 5

## Figures and Tables

**Table 1:** Disease characteristics and medical treatments.

Control	Age	Sex						
02C	69	F						
04C	69	F						
05C	64	F						
07C	65	M						
09C	47	F						
10C	53	F						
11C	45	F						
12C	65	M						
14C	65	M						
Psoriasis patients			**Ao**	**Psa**	**Medication**	**Topical treatment**	**PASI**	**Type and severity**
02P	62	F	17	0	0	0	9.0	small plaque, moderate
03P	64	M	9	0	adalimumab	mometasone	13.3	plaque, severe
05P	65	F	6	0	0	betametasone/salisylic acid, calcipotriene	10.0	plaque, guttate, moderate
06P	20	M	15	1	0	calcipotriene/betamethasone	14.7	plaque, moderate
08P	50	M	25	1	methotrexate	0	14.8	plaque, guttate, severe
09P	60	M	30	0	0	calcipotriene/betamethasone	11.9	plaque, moderate

Abbreviations: 0, No; 1, Yes; F, female; M, male; Psa, Psoriasis arthritis; PASI, Psoriasis Area Severity Index; Ao, Age of onset.

**Table 2:** NLR (A), RLR (B), cytoplasmic DNA-sensing (C) pathway components, and other inflammasome-related genes (D) that show upregulation in lesions (GW-PLvsPN).

Symbol	Gene name	FC	FDR	A	B	C	D	MitsuiFC^18^	Li FC^9^
AIM2	absent in melanoma 2	4.6E + 08	0.00			x			6.4
ATG5	autophagy related 5	5.3	4.19		x				0.9
AZI2	5-azacytidine induced 2	1.5	0.50		x				1.2
BIRC3	baculoviral IAP repeat containing 3	2.8	1.76	x					2.4
CAMP	cathelicidin antimicrobial peptide	5.6E + 09	0.00			x	x		7.7
CARD6	caspase recruitment domain family, member 6	1.0E + 09	0.00	x					3.3
CARD18	caspase recruitment domain family, member 18	1.1E + 09	0.34	x					1.3
CASP1	caspase 1, apoptosis-related cysteine peptidase	3.0	0.00	x		x		2.4	1.5
CASP5	caspase 5, apoptosis-related cysteine peptidase	7.6E + 08	0.00	x					9.8
CASP8	caspase 8, apoptosis-related cysteine peptidase	3.1	2.74	x	x				1.5
CASP10	caspase 10, apoptosis-related cysteine peptidase	2.5	0.41		x				1.6
CCL4	chemokine ligand 4	2.2E + 09	0.00			x			5.3
CXCL1	chemokine ligand 1	5.6E + 08	0.00	x				8.0	70.6
CYLD	cylindromatosis (turban tumor syndrome)	2.2	2.89		x			3.3	1.2
DDX3X	DEAD box polypeptide 3, X-linked	1.7	1.74		x			2.6	1.1
DDX58	DEAD box polypeptide 58	3.0	0.23	x	x	x		2.9	2.5
HSP90B1	heat shock protein 90kDa beta (Grp94), 1	2.0	4.98	x					1.1
IFI16	interferon, gamma-inducible protein 16	2.4	0.23			x	x		2.4
IFIH1	interferon induced with helicase C domain 1	4.3E + 08	0.26		x				2.8
IKBKE	inhibitor of k light pp enhancer in Bcells, kinase e	1.9	0.57		x	x			2.0
IL1B	interleukin 1, beta	1.1E + 09	0.00	x		x			6.8
IL8	interleukin 8	7.3E + 09	0.00	x	x			7.4	344.2
IRF7	interferon regulatory factor 7	2.3E + 08	1.65		x	x		2.8	5.4
ISG15	ISG15 ubiquitin-like modifier	7.2	0.00		x			4.4	5.5
MAPK8	mitogen-activated protein kinase 8	7.2E + 08	0.07	x	x				1.1
MAPK13	mitogen-activated protein kinase 13	2.9	0.23	x	x			2.4	1.5
NLRP10	NLR family, pyrin domain containing 10	3.5	0.96	x			x		NA
NLRX1	NLR family member X1	5.9	0.00		x		x	2.5	2.2
NOD2	nucleotide-binding oligomerization domain containing 2	6.0	0.00	x				3.4	3.1
		4.3E + 08	0.75	x					
POLR3B	polymerase (RNA) III polypeptide B	2.3	1.30			x			1.3
POLR3G	polymerase (RNA) III polypeptide G (32kD)	5.7	0.00			x		2.8	4.3
PYCARD	PYD and CARD domain containing	4.1	1.98	x		x		2.1	2.0
3.0	0.31	x		x					
PYDC1	PYD (pyrin domain) containing 1	3.6	0.16	x					2.0
SUGT1	SGT1, suppressor of G2 allele of SKP1	1.6	0.42	x					1.2
TAB1	TGF-b activated kinase 1/MAP3K7 binding prot 1	1.60	4.67	x					0.8
TANK	TRAF family member-associated NFKB activator	1.73	0.16		x			2.1	1.0
TRIM25	tripartite motif containing 25	2.61	0.75		x				1.6

Abbreviations: TFE, Transcript First Exon; FC, Fold Change; FDR, False Discovery Rate (%).
