# Supplementary material for: NOD-like receptor signaling and inflammasome-related pathways are highlighted in psoriatic epidermis
Source: Sci Rep. 2016 Mar 15;6:22745. doi: 10.1038/srep22745 (PMC4792137; doi:10.1038/srep22745)
Supplement: Supplementary Information [file srep22745-s1.pdf]

Supplementary Information

NOD-like receptor signaling and inflammasome-related pathways are highlighted in psoriatic epidermis

Mari H. Tervaniemi, Shintaro Katayama, Tiina Skoog, H. Annika Siitonen, Jyrki Vuola, Kristo Nuutila, Raija Sormunen, Anna Johnsson, Sten Linnarsson, Sari Suomela, Esko Kankuri, Juha Kere, and Outi Elomaa

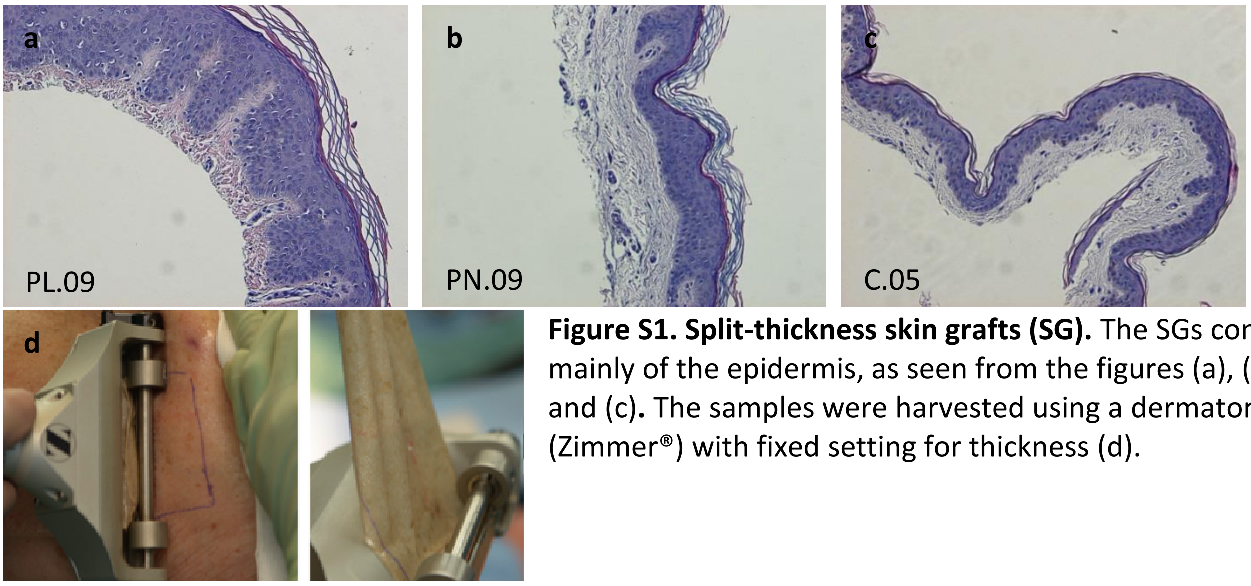

Figure S1. Split-thickness skin grafts (SG). The SGs consist mainly of the epidermis, as seen from the figures (a), (b), and (c)**.** The samples were harvested using a dermatome (Zimmer®) with fixed setting for thickness (d).

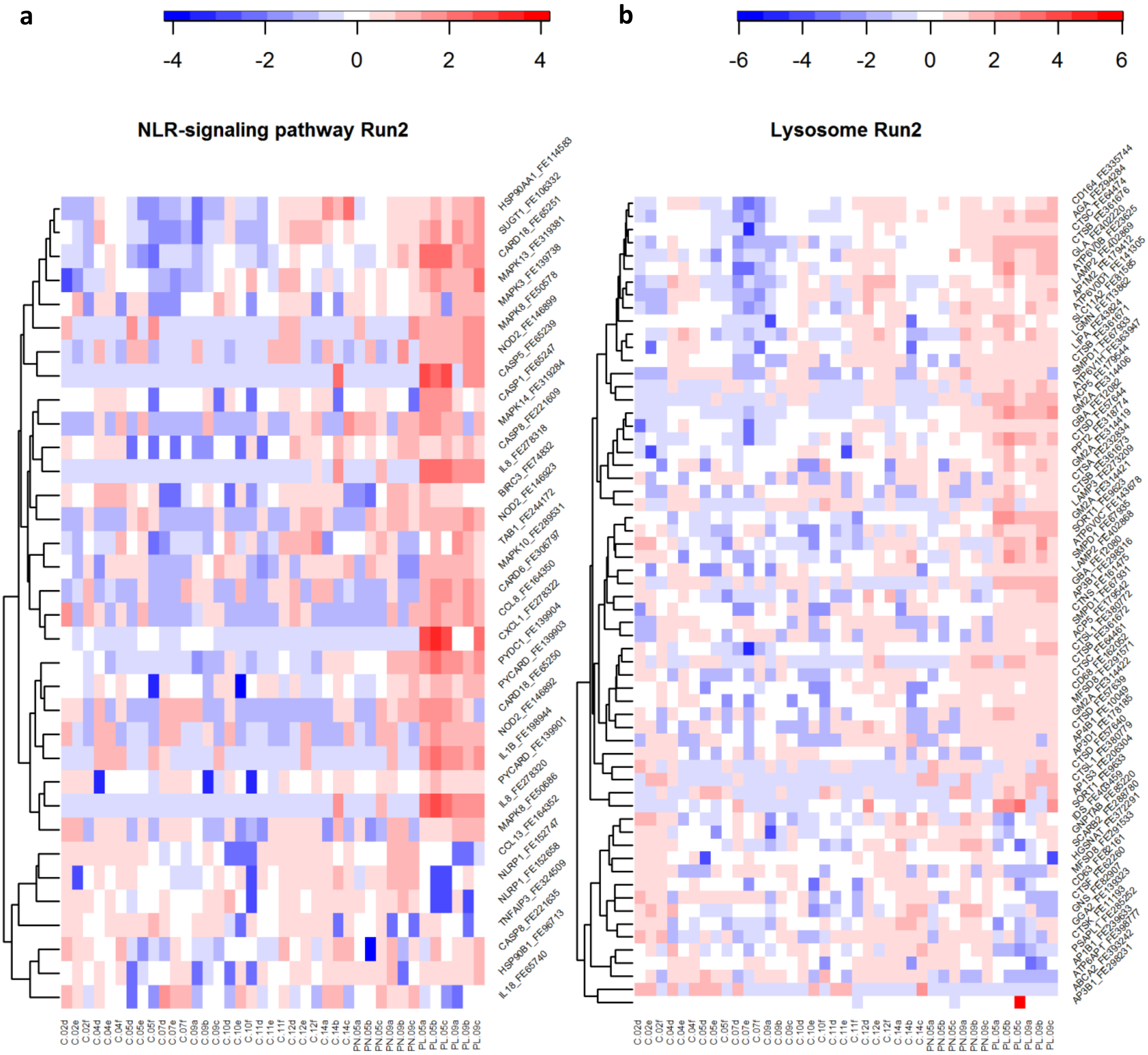

c

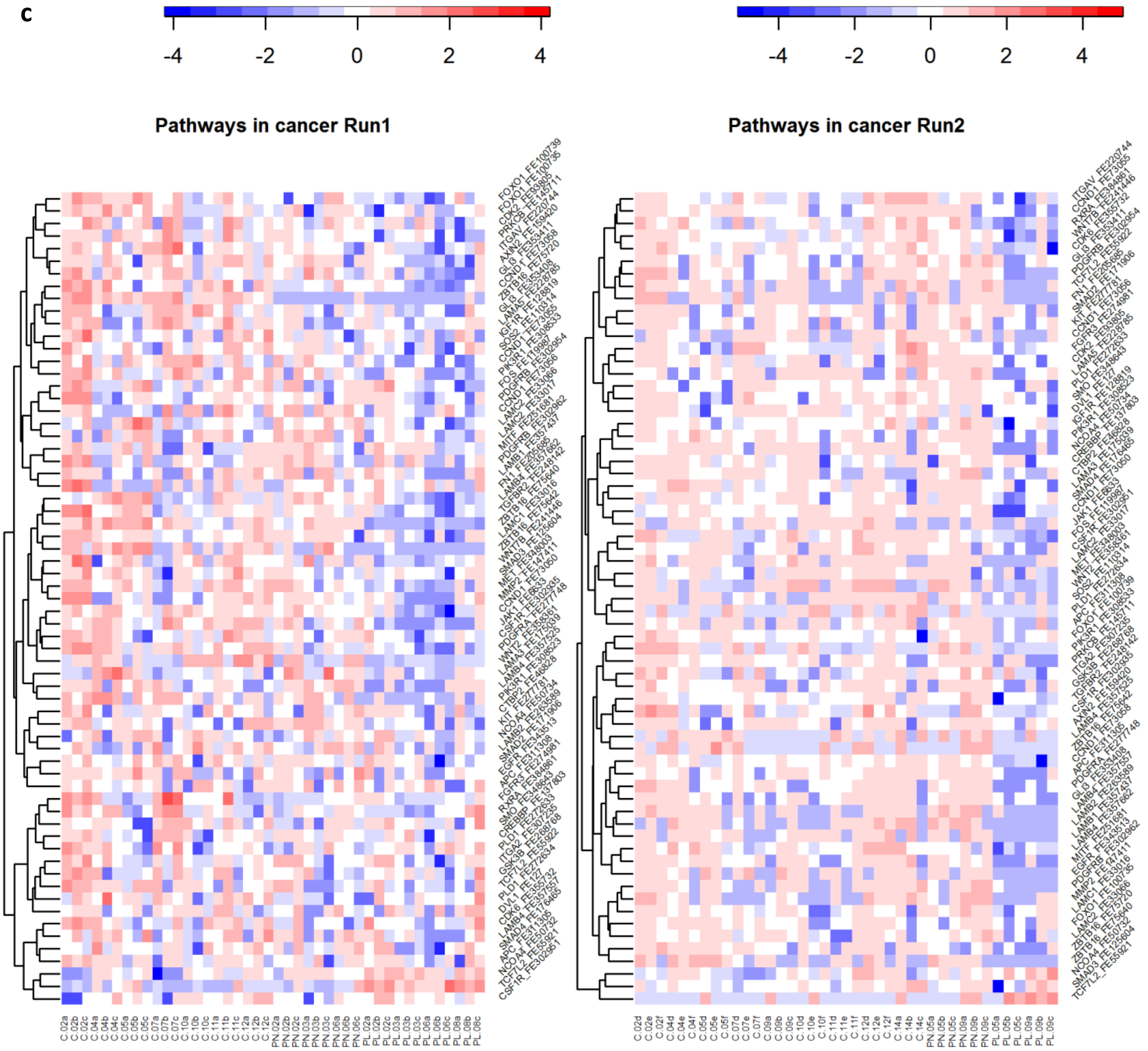

Figure S2. Gene expression profiles of up- and downregulated pathways in psoriatic lesions. Heat maps of genes involved in the NLR signaling (Run2; Run1 shown in Figure 1) (a), lysosome (Run2; Run1 shown in Figure 1) (b), and pathways in cancer (c). Lysosome includes several V-ATPase and cathepsin genes that are upregulated in PL samples. Pathways in cancer is identifiable among both the upregulated and downregulated DEGs of PL. The heat map (c) shows only the downregulated genes. Color key: red represents upregulation and blue downregulation, according to the intensity of the color (row Z-score).

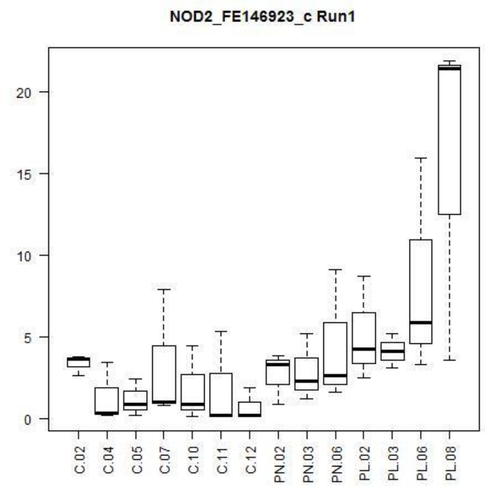
NOD2_FE146923_c Run1
0
5
10
15
20
C.02
C.04
C.05
C.07
C.10
C.11
C.12
PN.02
PN.03
PN.06
PL.02
PL.03
PL.06
PL.08

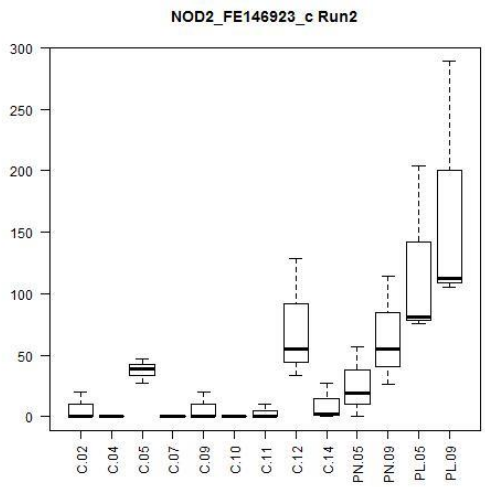
NOD2_FE146923_c Run2
0
50
100
150
200
250
300
C.02
C.04
C.05
C.07
C.09
C.10
C.11
C.12
C.14
PN.05
PN.09
PL.05
PL.09

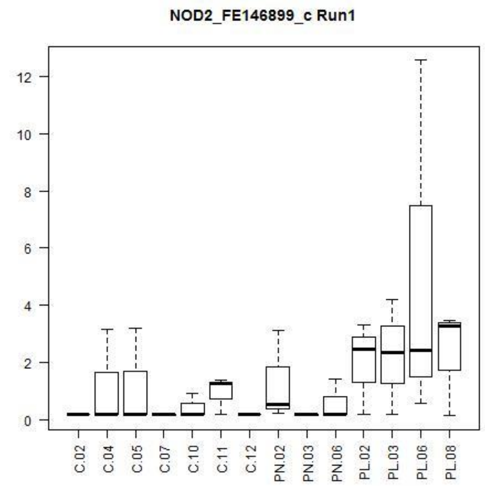
NOD2_FE146899_c Run1
0
2
4
6
8
10
12
C.02
C.04
C.05
C.07
C.10
C.11
C.12
PN.02
PN.03
PN.06
PL.02
PL.03
PL.06
PL.08

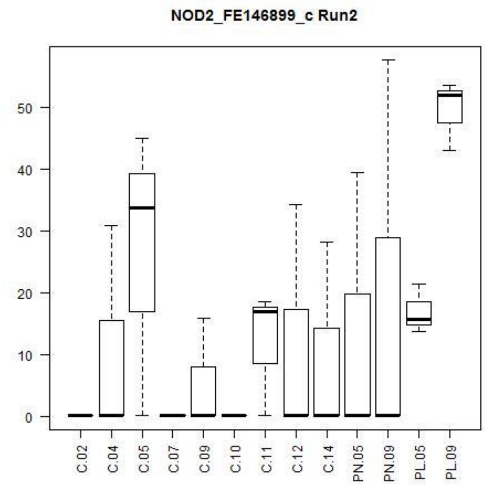
NOD2_FE146899_c Run2
0
10
20
30
40
50
C.02
C.04
C.05
C.07
C.09
C.10
C.11
C.12
C.14
PN.05
PN.09
PL.05
PL.09

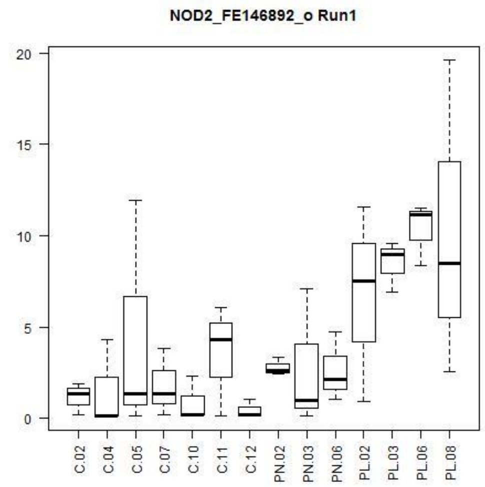
NOD2_FE146892_o Run1
0
5
10
15
20
C.02
C.04
C.05
C.07
C.10
C.11
C.12
PN.02
PN.03
PN.06
PL.02
PL.03
PL.06
PL.08

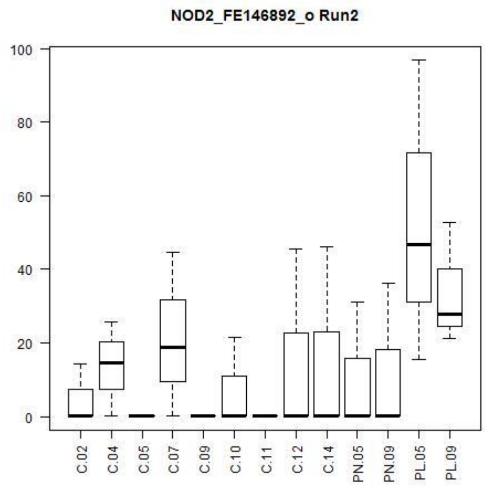
NOD2_FE146892_o Run2
0
20
40
60
80
100
C.02
C.04
C.05
C.07
C.09
C.10
C.11
C.12
C.14
PN.05
PN.09
PL.05
PL.09

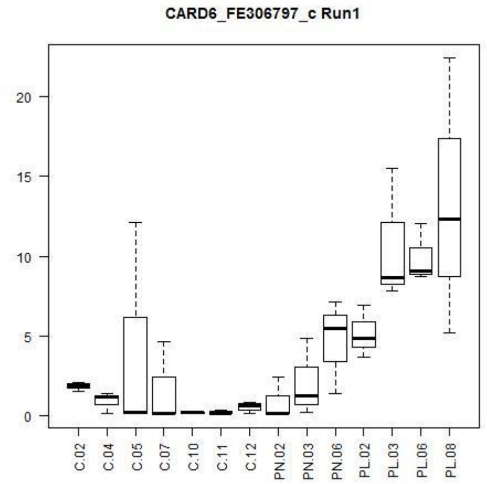
CARD6_FE306797_c Run1
0
5
10
15
20
C.02
C.04
C.05
C.07
C.10
C.11
C.12
PN.02
PN.03
PN.06
PL.02
PL.03
PL.06
PL.08

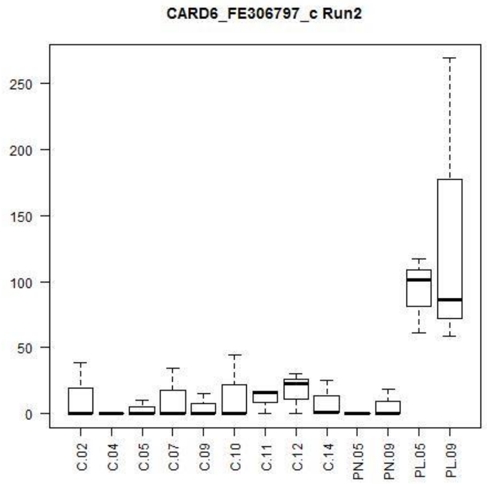
CARD6_FE306797_c Run2
0
50
100
150
200
250
C.02
C.04
C.05
C.07
C.09
C.10
C.11
C.12
C.14
PN.05
PN.09
PL.05
PL.09

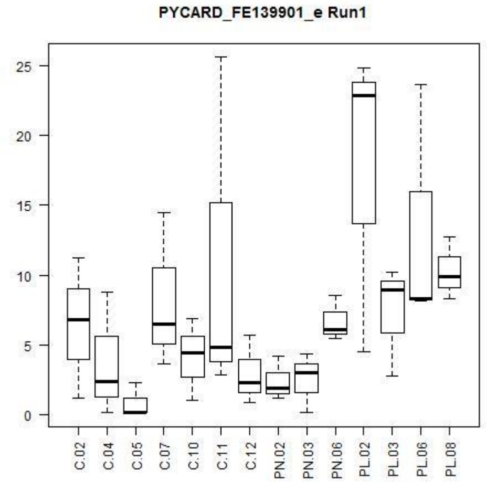
PYCARD_FE139901_e Run1
0
5
10
15
20
25
C.02
C.04
C.05
C.07
C.10
C.11
C.12
PN.02
PN.03
PN.06
PL.02
PL.03
PL.06
PL.08

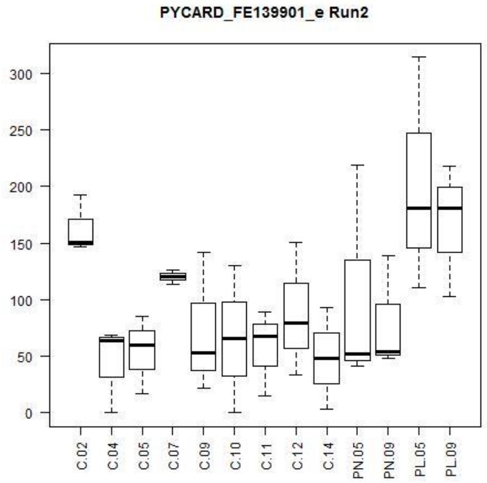
PYCARD_FE139901_e Run2
0
50
100
150
200
250
300
C.02
C.04
C.05
C.07
C.09
C.10
C.11
C.12
C.14
PN.05
PN.09
PL.05
PL.09

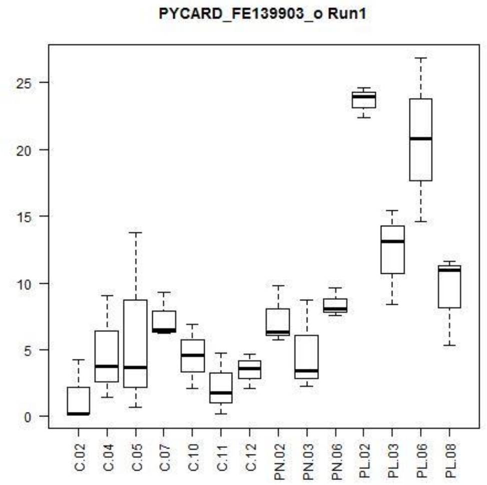
PYCARD_FE139903_o Run1
0
5
10
15
20
25
C.02
C.04
C.05
C.07
C.10
C.11
C.12
PN.02
PN.03
PN.06
PL.02
PL.03
PL.06
PL.08

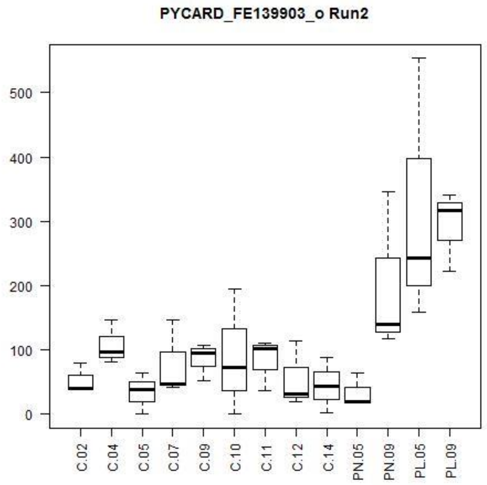
PYCARD_FE139903_o Run2
0
100
200
300
400
500
C.02
C.04
C.05
C.07
C.09
C.10
C.11
C.12
C.14
PN.05
PN.09
PL.05
PL.09

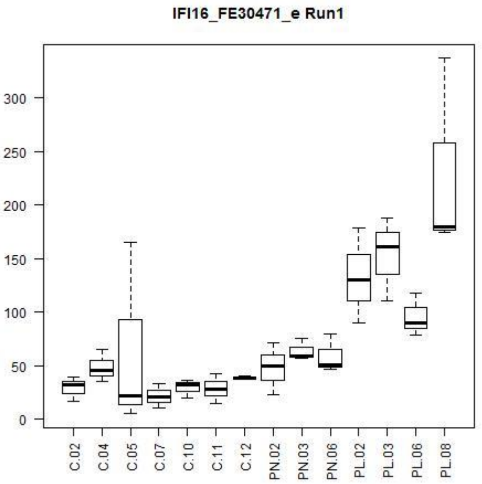
IFI16_FE30471_e Run1
0
50
100
150
200
250
300
C.02
C.04
C.05
C.07
C.10
C.11
C.12
PN.02
PN.03
PN.06
PL.02
PL.03
PL.06
PL.08

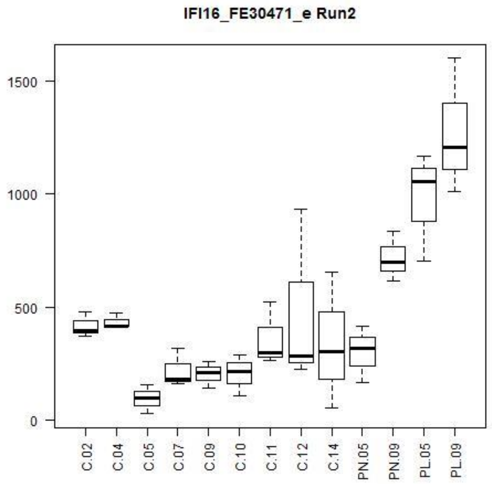
IFI16_FE30471_e Run2
0
500
1000
1500
C.02
C.04
C.05
C.07
C.09
C.10
C.11
C.12
C.14
PN.05
PN.09
PL.05
PL.09

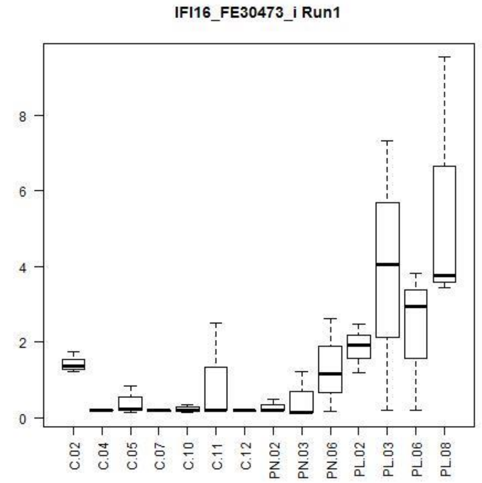
IFI16_FE30473_i Run1
0
2
4
6
8
C.02
C.04
C.05
C.07
C.10
C.11
C.12
PN.02
PN.03
PN.06
PL.02
PL.03
PL.06
PL.08

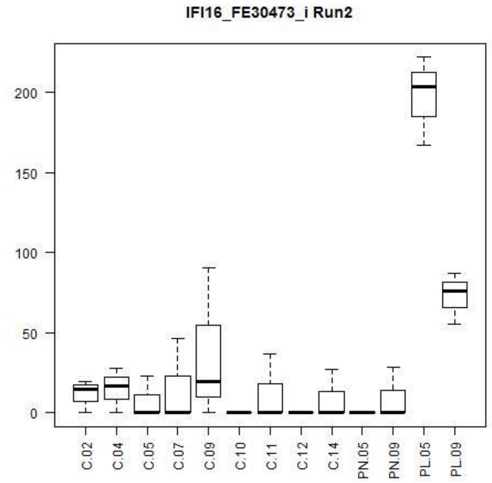
IFI16_FE30473_i Run2
0
50
100
150
200
C.02
C.04
C.05
C.07
C.09
C.10
C.11
C.12
C.14
PN.05
PN.09
PL.05
PL.09

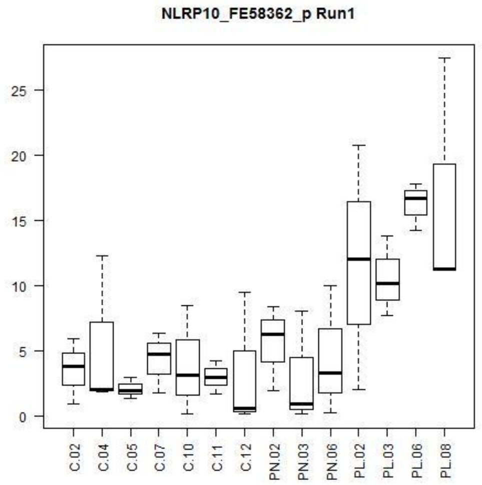
NLRP10_FE58362_p Run1
0
5
10
15
20
25
C.02
C.04
C.05
C.07
C.10
C.11
C.12
PN.02
PN.03
PN.06
PL.02
PL.03
PL.06
PL.08

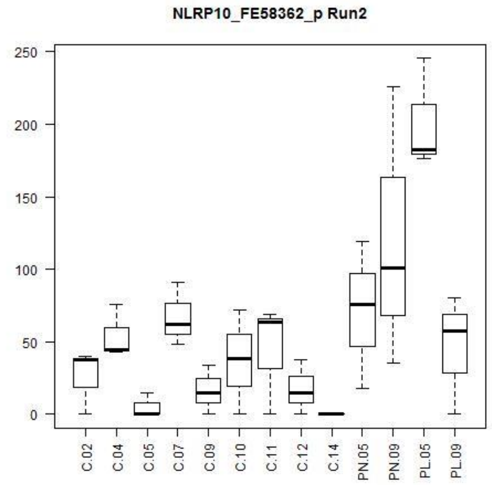
NLRP10_FE58362_p Run2
0
50
100
150
200
250
C.02
C.04
C.05
C.07
C.09
C.10
C.11
C.12
C.14
PN.05
PN.09
PL.05
PL.09

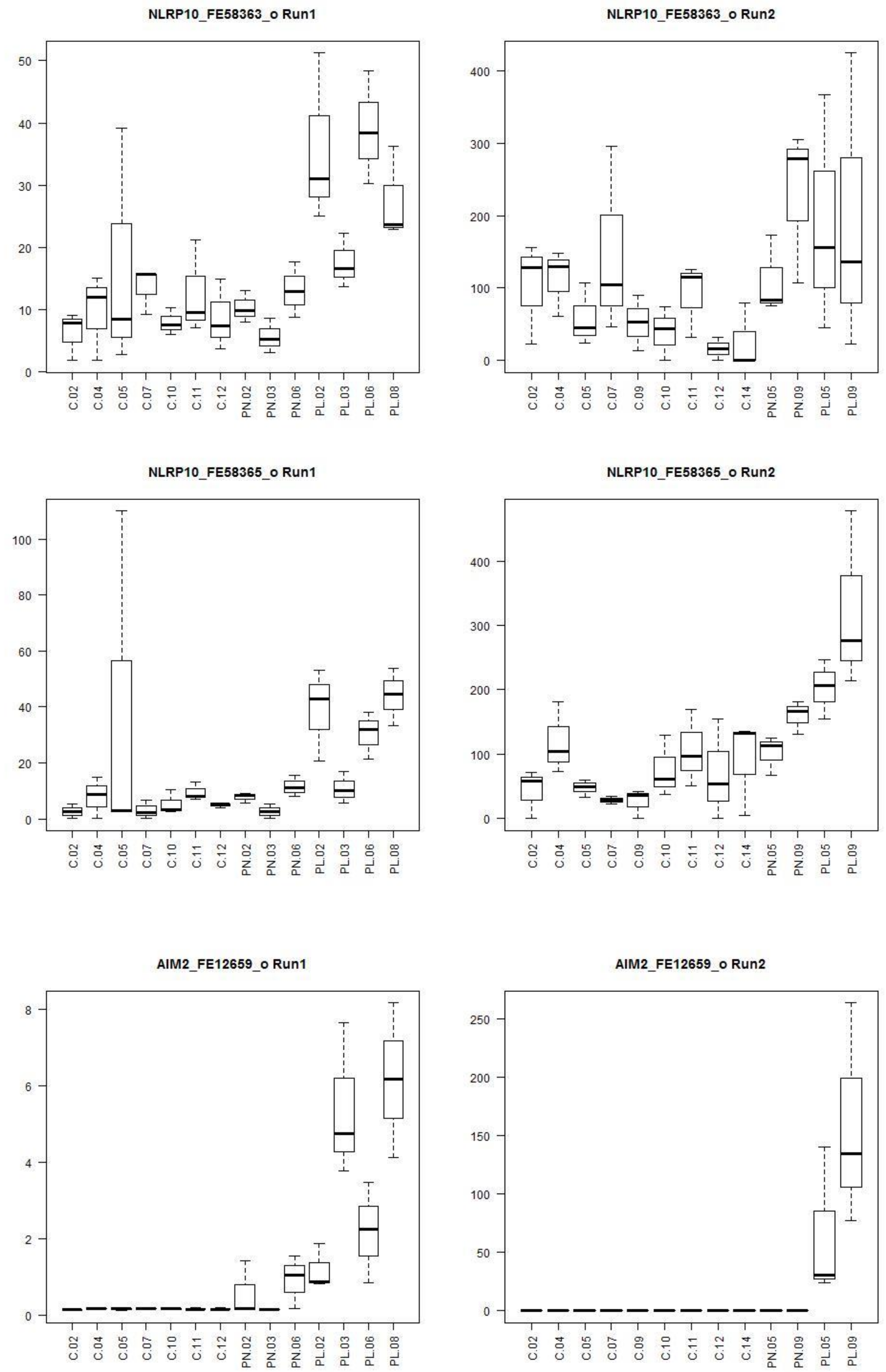
NLRP10_FE58363_o Run1
0
10
20
30
40
50
C.02 C.04 C.05 C.07 C.10 C.11 C.12 PN.02 PN.03 PN.06 PL.02 PL.03 PL.06 PL.08
NLRP10_FE58363_o Run2
0
100
200
300
400
C.02 C.04 C.05 C.07 C.09 C.10 C.11 C.12 C.14 PN.05 PN.09 PL.05 PL.09
NLRP10_FE58365_o Run1
0
20
40
60
80
100
C.02 C.04 C.05 C.07 C.10 C.11 C.12 PN.02 PN.03 PN.06 PL.02 PL.03 PL.06 PL.08
NLRP10_FE58365_o Run2
0
100
200
300
400
C.02 C.04 C.05 C.07 C.09 C.10 C.11 C.12 C.14 PN.05 PN.09 PL.05 PL.09
AIM2_FE12659_o Run1
0
2
4
6
8
C.02 C.04 C.05 C.07 C.10 C.11 C.12 PN.02 PN.03 PN.06 PL.02 PL.03 PL.06 PL.08
AIM2_FE12659_o Run2
0
50
100
150
200
250
C.02 C.04 C.05 C.07 C.09 C.10 C.11 C.12 C.14 PN.05 PN.09 PL.05 PL.09

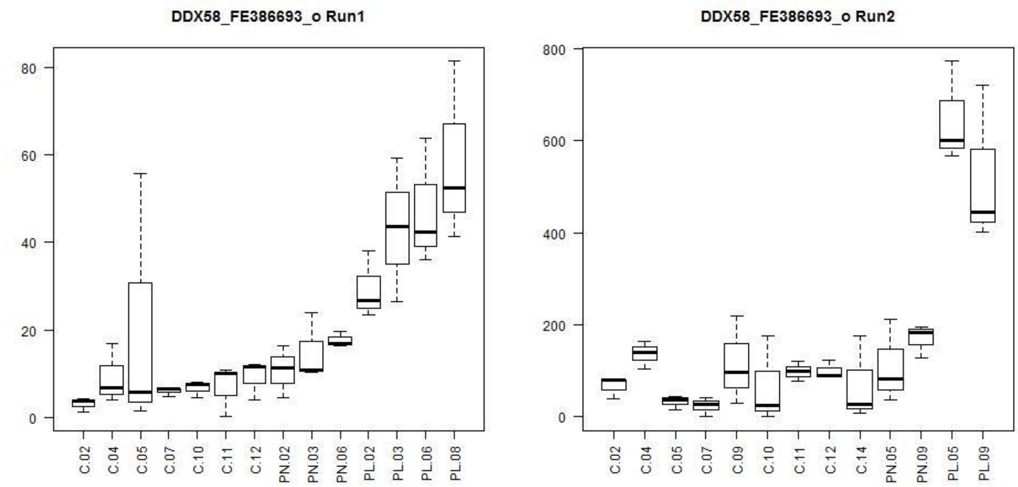

Figure S3. Expression of the NLR-pathway key component transcripts shown in box plots. Plots are based on the spike-in normalized gene expression data of RNAseq.

a

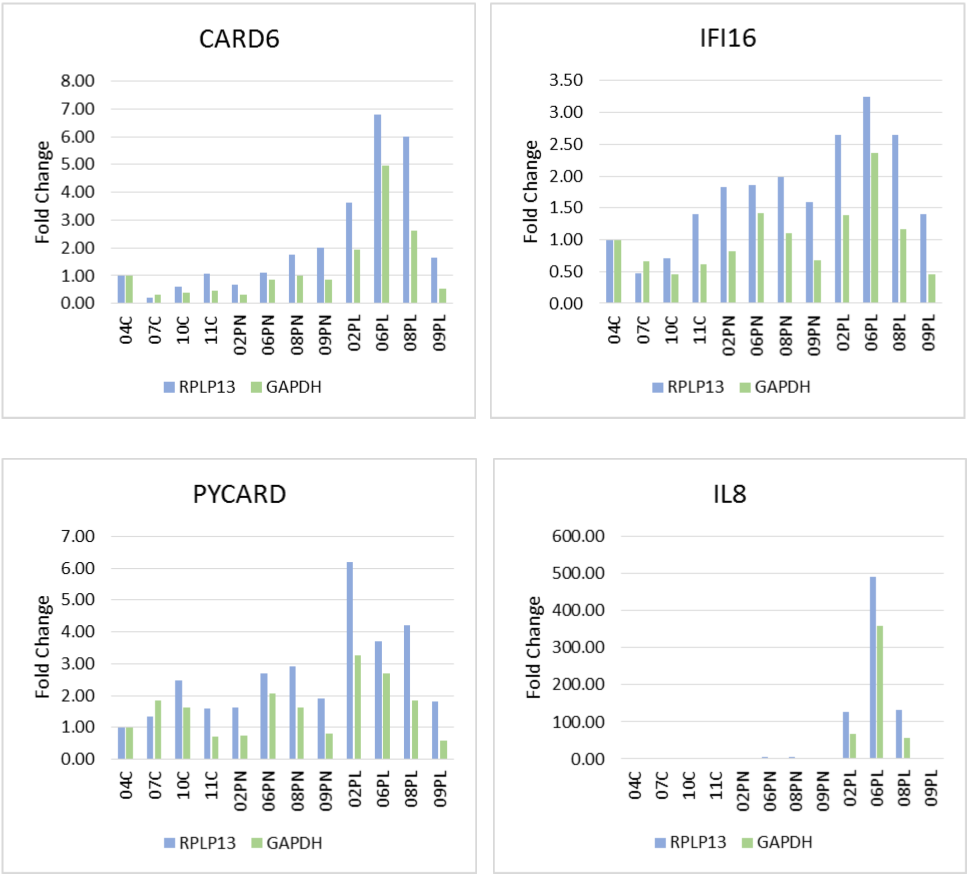

qPCR primers:	Forward	Reverse
CARD6	AGGCGACCTGTCAGCATTTT	TTAAAACTTCATGCCTTAAGCCGC
IFI16	AACCACGTTGAAACCAAGACT	TGCGTTCAGCACCATCACTT
PYCARD	GTTCAAGCTGAAGCTGCTGTCG	GTGAGGTCCAAGGCGTCCAT
IL8	TCTGTGTGAAGGTGCAGTTTTG	TGGGGTGGAAAGGTTTGGAGT
RPLP13	CTCAAGGTCGTGCGTCTGAA	TGGCTGTCACTGCCTGGTACT
GAPDH	GAGTCAACGGATTTGGTCGT	CATGGGTGGAATCATATTGG

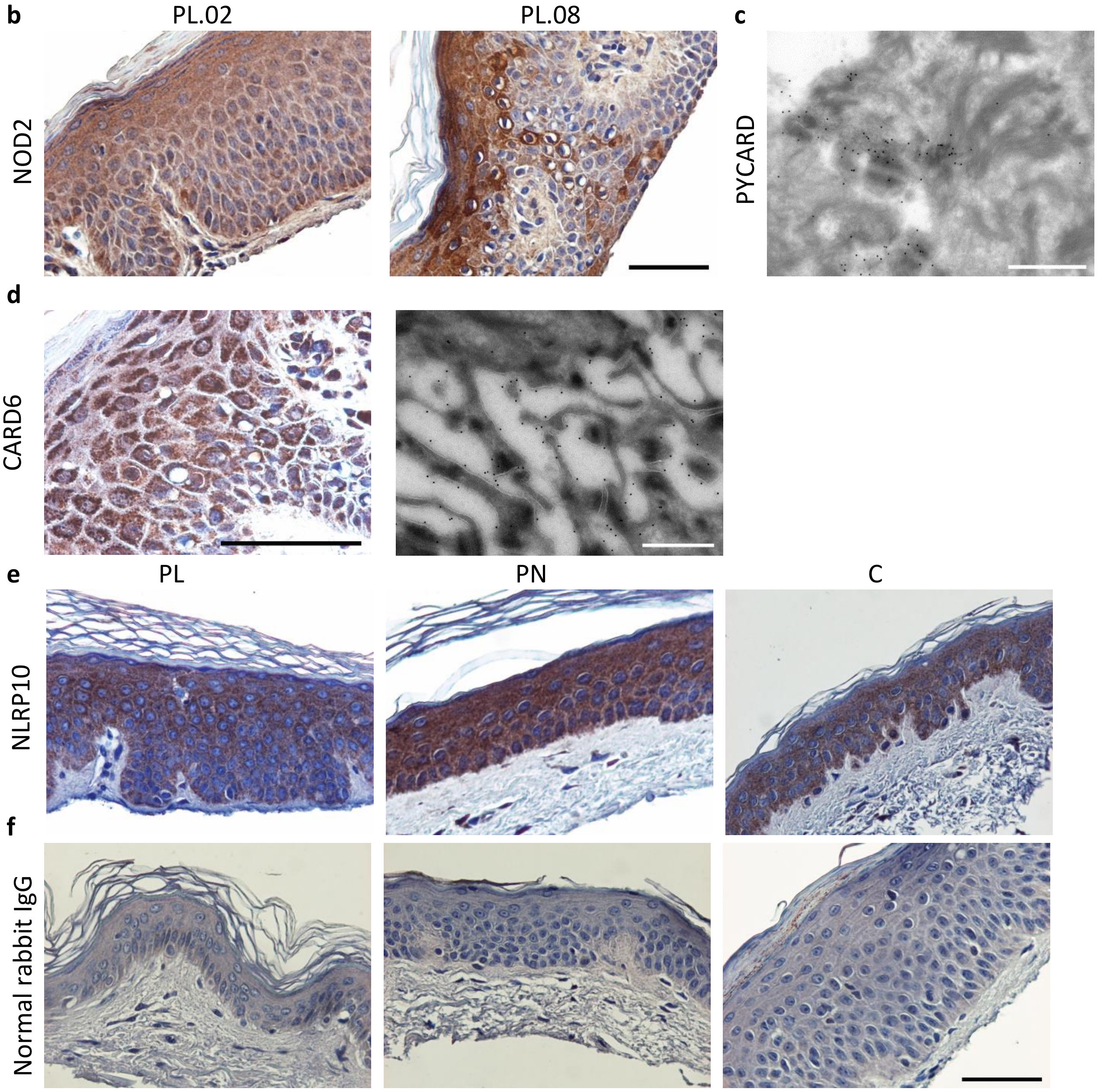

Figure S4. Expression of the NLR-signaling related genes in psoriatic and control epidermis. (a) Confirmation of changes in the expression of *CARD6*, *IFI16*, *PYCARD*, and *IL8*, with qPCR. Two control genes were used for normalization: RPLP13 and GAPHD. The samples were compared to the sample C.04, which therefore has the value 1. (b) NOD2 has induced expression in PL epidermis, which varies between individuals: staining in PL.02 sample is moderate whereas in PL.08 it is highly upregulated when compared with corresponding PN samples. (c) IEM of PL skin shows PYCARD clusters in connection with membrane structures that resemble desmosomes. (d) In the higher magnification of immunostaining in PL skin CARD6 is observable as cytoplasmic granules. The IEM reveals abundant CARD6 in keratinocyte contacts as well. (e) Immunohistochemistry (IH) with antibody against NLRP10 reveals strong expression in control as well as in psoriatic skin (PL and PN). (f) Control stainings with normal rabbit IgG are negative. IH scale bars: 50µm. IEM scale bars: 500nm.

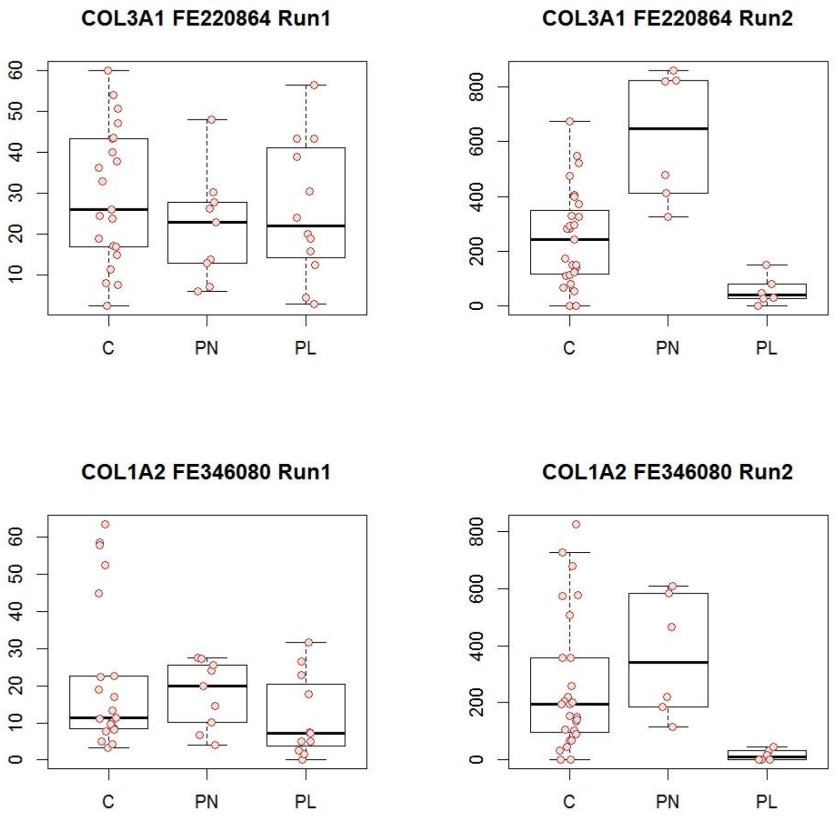

Figure S5. Expression of the fibroblast marker genes shown in box plots. Plots are based on the spike-in normalized gene expression data of RNAseq. The Run1 contains samples: 02C, 04C, 05C, 07C, 10C, 11C, 12C, 02PN, 03PN, 06PN, 02PL, 03PL, 06PL, and 08PL. The Run2 contains samples: 02C, 04C, 05C, 07C, 09C, 10C, 11C, 12C, 14C, 05PN, 09PN, 05PL, and 09PL. All the triplicates of each sample are shown as pink spots. We selected the fibroblast markers *COL3A1* and *COL1A2* and examined their expression. We could see a slight decrease in the expression of these markers in some of the samples, which might be due to the thickened architecture of the epidermis.

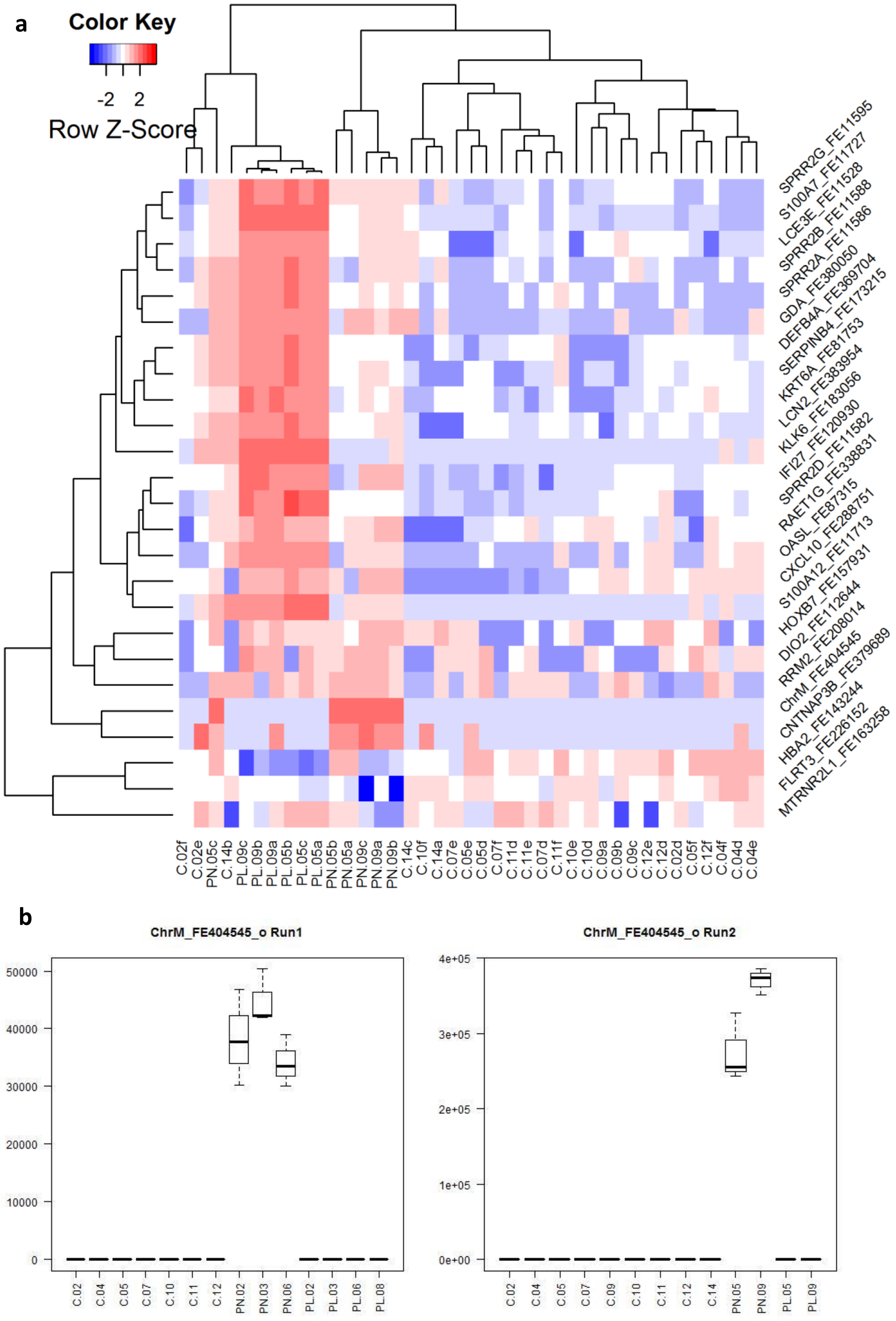
a
Color Key
-2 2
Row Z-Score
SPRR2G_FE11595
S100A7_FE11727
LCE3E_FE11528
SPRR2B_FE11588
SPRR2A_FE11586
GDA_FE380050
DEFB4A_FE369704
SERPINB4_FE173215
KRT6A_FE81753
LCN2_FE383954
KLK6_FE183056
IFI27_FE120930
SPRR2D_FE11582
RAET1G_FE338831
OASL_FE87315
CXCL10_FE288751
S100A12_FE11713
HOXB7_FE157931
DIO2_FE112644
RRM2_FE208014
ChrM_FE404545
CNTNAP3B_FE379689
HBA2_FE143244
FLRT3_FE226152
MTRNR2L1_FE163258
C.02f
C.02e
PN.05c
C.14b
PL.09c
PL.09b
PL.09a
PL.05b
PL.05c
PL.05a
PN.05b
PN.05a
PN.09c
PN.09a
PN.09b
C.14c
C.10f
C.14a
C.07e
C.05e
C.05d
C.07f
C.11d
C.11e
C.07d
C.11f
C.10e
C.10d
C.09a
C.09b
C.09c
C.12e
C.12d
C.02d
C.05f
C.12f
C.04f
C.04d
C.04e
b
ChrM_FE404545_o Run1
50000
40000
30000
20000
10000
0
C.02
C.04
C.05
C.07
C.10
C.11
C.12
PN.02
PN.03
PN.06
PL.02
PL.03
PL.06
PL.08
ChrM_FE404545_o Run2
4e+05
3e+05
2e+05
1e+05
0e+00
C.02
C.04
C.05
C.07
C.09
C.10
C.11
C.12
C.14
PN.05
PN.09
PL.05
PL.09

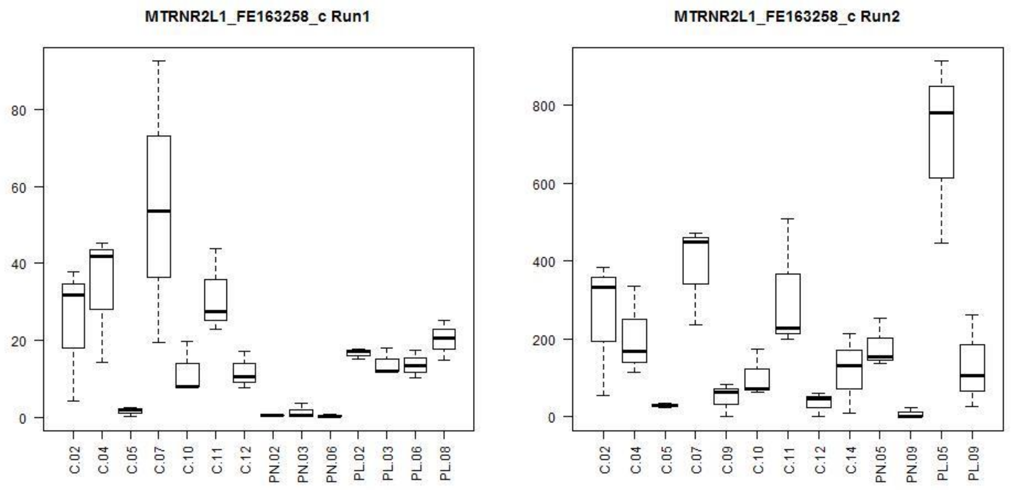

c

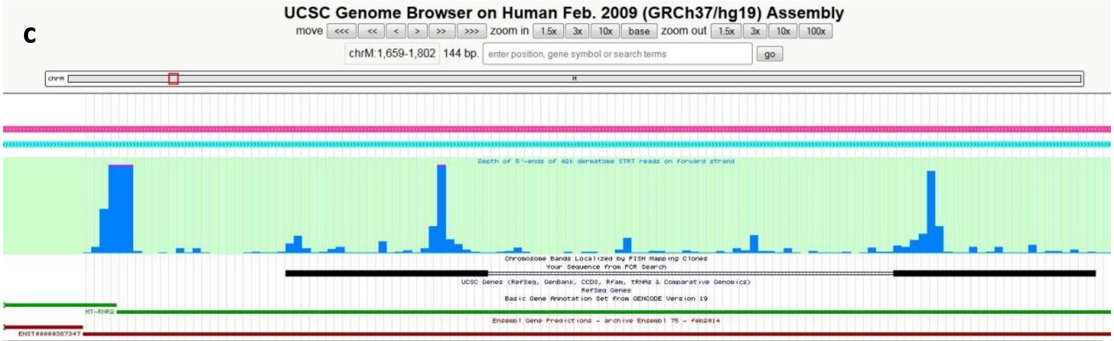

chrM:1673 → GCTAAACCTAGCCCCAAACCCACTCCACCTTACTACCAG (~ 95% of total alignments at this site)

d

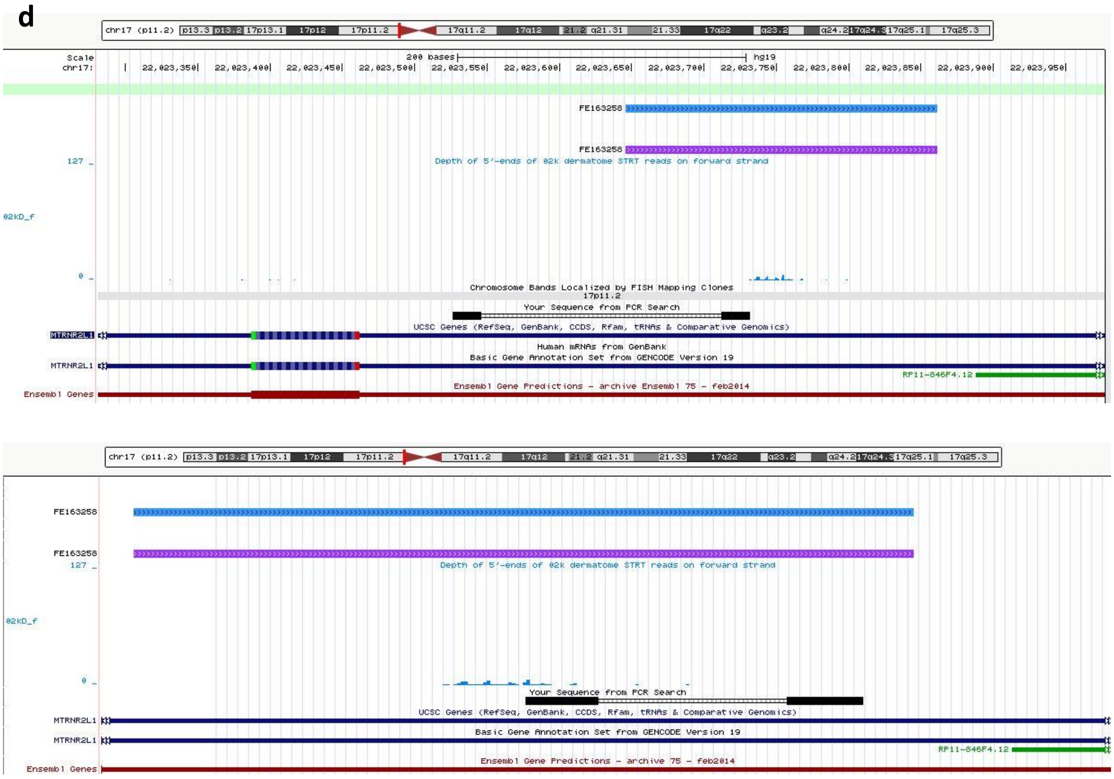

e qPCR primers:

	Forward	Reverse
MTRNR2 (FE404545)	CACTCCACCTTACTACCAGACAAC	GGTACTATATCTATTGCGCCAGGT
MTRNR2L1 (FE163258)	AGGACATCCCAATGGTGCAG	ACCTGGATTACTCCGGTCTGA
RNA18S5	GTGAAACTGCGAATGGCTC	CGTCGGCATGTATTAGCTC
Mottaghi-Dastjerdi et al. 2014:		
MTRNR2L1 (FE163258)	CGCAGGCCCTAAACTACCAG	TGCTACTGTCGATGTGGACC

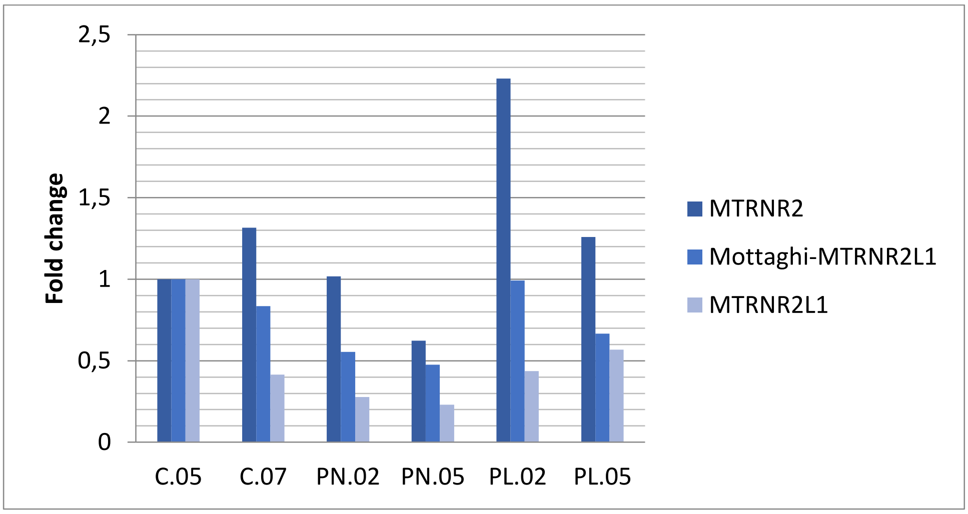

Figure S6: Differentially expressed genes in non-lesional psoriatic skin. (a) Heat map of DEGs from the PNvsC comparison; gene expressions in samples (PL, PN, and C) of the Run2. Color key: red represents upregulated and blue downregulated expression levels according to the intensity of the color (row Z-score). The gene name includes transcript first exon (TFE) code. The most highly upregulated transcript in PN samples, ChrM_FE404545, represents MTRNR2 and shows no induction in PL. Heat map of the Run1 is shown in Figure 5. (b) Box plots of ChrM_FE404545_o and MTRNR2L1_FE163258_c. Plots are based on the spike-in normalized gene expression data of RNAseq. (c) Chromosomal localization of C.02 reads shown in UCSC genome browser: ChrM_FE404545_o reads map at the start site of the MTRNR2 gene and (d) MTRNR2L1 reads align with the 3'-end region of the gene. (e) MTRNR2 and MTRNR2L1 expressions were studied in SG samples (PL, PN, and C) by qPCR. Here presented data from a representative experiment where RNA18S5 was used as a reference and the data compared here to C.05 (PCR target regions shown in c and d). We were unable to detect significant difference between the sample groups with qPCR.
